# Varied Length Stokes Shift BODIPY-Based Fluorophores for Multicolor Microscopy

**DOI:** 10.1038/s41598-018-22892-8

**Published:** 2018-03-15

**Authors:** Amy M. Bittel, Ashley M. Davis, Lei Wang, Michel A. Nederlof, Jorge O. Escobedo, Robert M. Strongin, Summer L. Gibbs

**Affiliations:** 1 Biomedical Engineering Department, Oregon Health & Science University, Portland, OR 97201 USA; 2 Knight Cancer Institute, Oregon Health & Science University, Portland, OR 97201 USA; 3 OHSU Center for Spatial Systems Biomedicine, Oregon Health & Science University, Portland, OR 97201 USA; 4 Quantitative Imaging, Pittsburgh, PA 15238 USA; 5 Department of Chemistry, Portland State University, Portland, OR 97201 USA

## Abstract

Multicolor microscopy tools necessary to localize and visualize the complexity of subcellular systems are limited by current fluorophore technology. While commercial fluorophores cover spectral space from the ultraviolet to the near infrared region and are optimized for conventional bandpass based fluorescence microscopy, they are not ideal for highly multiplexed fluorescence microscopy as they tend to have short Stokes shifts, restricting the number of fluorophores that can be detected in a single sample to four to five. Herein, we synthesized a library of 95 novel boron-dipyrromethene (BODIPY)-based fluorophores and screened their photophysical, optical and spectral properties for their utility in multicolor microscopy. A subset of our BODIPY-based fluorophores yielded varied length Stokes shifts probes, which were used to create a five-color image using a single excitation with confocal laser scanning microscopy for the first time. Combining these novel fluorophores with conventional fluorophores could facilitate imaging in up to nine to ten colors using linear unmixing based microscopy approaches.

## Introduction

Multicolor microscopy has gained in popularity for the detection and visualization of multiple subcellular entities in a single sample. Specificity in highly multiplexed microscopy routinely relies on small molecule fluorophore tagged affinity reagents such as antibodies, antibody fragments, nanobodies, affibodies, and lectins for specific biomolecular labeling. Imaging using conventional bandpass based microscopy is common, where spectral separation is limited to four to five colors that span the ultraviolet (UV) to near-infrared (NIR) spectra. Unique fluorophore detection in a single sample can be improved using spectral imaging and linear unmixing based fluorescence microscopy to simultaneously identify overlapping emission spectra, increasing multicolor imaging capability^1,2^. Fluorescence linear unmixing studies have demonstrated up to five color imaging with overlapping subcellular entities using two excitation wavelengths and emission distributed over 150 nm of spectral space^3^.

Optimal fluorophore technology for bandpassed based- and linear unmixing based-multicolor microscopy different substantially. Ideal fluorophores for bandpass-based multicolor microscopy have relatively short Stokes shifts (15–25 nm) and narrow emission spectra, minimizing spectral overlap for unique color detection. In contrast, fluorophores with varied length Stokes shifts are ideal for linear unmixing-based microscopy as they permit increased spectral detection using each excitation wavelength. Spectral separation using linear unmixing is also improved when emission spectra are distinct, where separate emission maxima and narrow or distinctly shaped emission spectra are desirable for a high degree of multiplexing^3^. Additionally, ideal fluorophores for both bandpass-based and linear unmixing-based multicolor microscopy generate high contrast, making it strategic to choose probes with high quantum yield and low signal to background ratio (SBR) for the untargeted small molecule fluorophore, permitting high SBR upon affinity reagent tagging^4^.

Imaging with fluorophore tagged affinity reagents enables versatility in fluorophore choice based on photophysical and optical properties, where affinity reagents are typically conjugated to small molecule fluorophores consisting of core chemical scaffolds including xanthene, rhodamine, Boron-dipyrromethene (BODIPY), cyanine, and oxazine^5,6^. While these fluorophore classes cover a broad range of excitation and emission spectra, they tend to have short Stokes shifts. For example, commercially available BODIPY based fluorophores are mostly limited to those with Stokes shifts less than 15 nm^5^. AlexaFluor dyes, one of the most popular brands of fluorophores, are based on a variety of scaffolds and have a typical Stokes Shift of 20 nm or less^5^. While these conventional fluorophores are ideal for bandpass-based multicolor imaging and have been used in for a variety of imaging studies, the need for complementary fluorophore technology optimized for linear unmixing-based multicolor microscopy persists. The development of a suite of fluorophores with both varied length Stokes shifts and the ability to generate high contrast would fulfill this need.

BODIPY is a widely utilized fluorophore scaffold due to its valuable photophysical and optical properties including its spectral stability at varied polarity, pH and physiologic conditions, resistance to photobleaching, relatively high quantum yield, and narrow emission spectra^7,8^. Furthermore, the BODIPY scaffold is particularly advantageous for novel fluorophore development since small modifications to its structure enables tuning of its fluorescence characteristics and the scaffold can be readily customized using a variety of synthetic routes^8–14^. Fluorophore libraries originating from various core BODIPY scaffolds have resulted in probes with an array of Stokes shifts which have demonstrated utility as chemical sensors and probes^15–18^. However, the previously synthesized BODIPY fluorophore libraries did not contain chemical moieties suitable for conjugation chemistries, limiting their utility for affinity tag labeling. Other BODIPY syntheses have successfully created long Stokes shift fluorophores for bioconjugation, but have been restricted to the NIR spectral region^19^.

Herein, we report the first synthesis of a BODIPY-based fluorophore library specifically designed to generate varied length Stokes shift probes that each contain a chemical moiety compatible with conjugation to affinity reagents for multicolor imaging applications. The novel BODIPY-based library was characterized for its SBR, photophysical, optical and spectral properties to enable selection of a subset of fluorophores most appropriate for linear unmixing-based multicolor microscopy imaging applications. The BODIPY-based library was also screened for organelle specificity in fixed, permeabilized cells and characterized for its *in vitro* biodistribution. Novel BODIPY-based fluorophores that targeted the nucleus and nucleolar regions, vesicular structures, and cytosolic regions in fixed cells were identified from the library. Through conjugation of our varied length Stokes shift BODIPY-based fluorophores to affinity reagents for five distinct subcellular structures, we demonstrated accurate labeling and imaging of five colors in a single sample with confocal laser scanning microscopy using spectral imaging and linear unmixing. Excitingly, our five-color image was obtained on spatially overlapping structures using a single excitation wavelength and emission spectra distributed over just 60 nm of spectral space. Utilization of our novel BODIPY-based fluorophores in combination with conventional short Stokes shift probes and other long Stokes shift fluorophores, such as the NIR BODIPY probes^12,19^, will permit highly multiplexed linear unmixing-based microscopy studies.

## Results

### Solid Phase Synthesis of BAA Library

**Figure 1:**
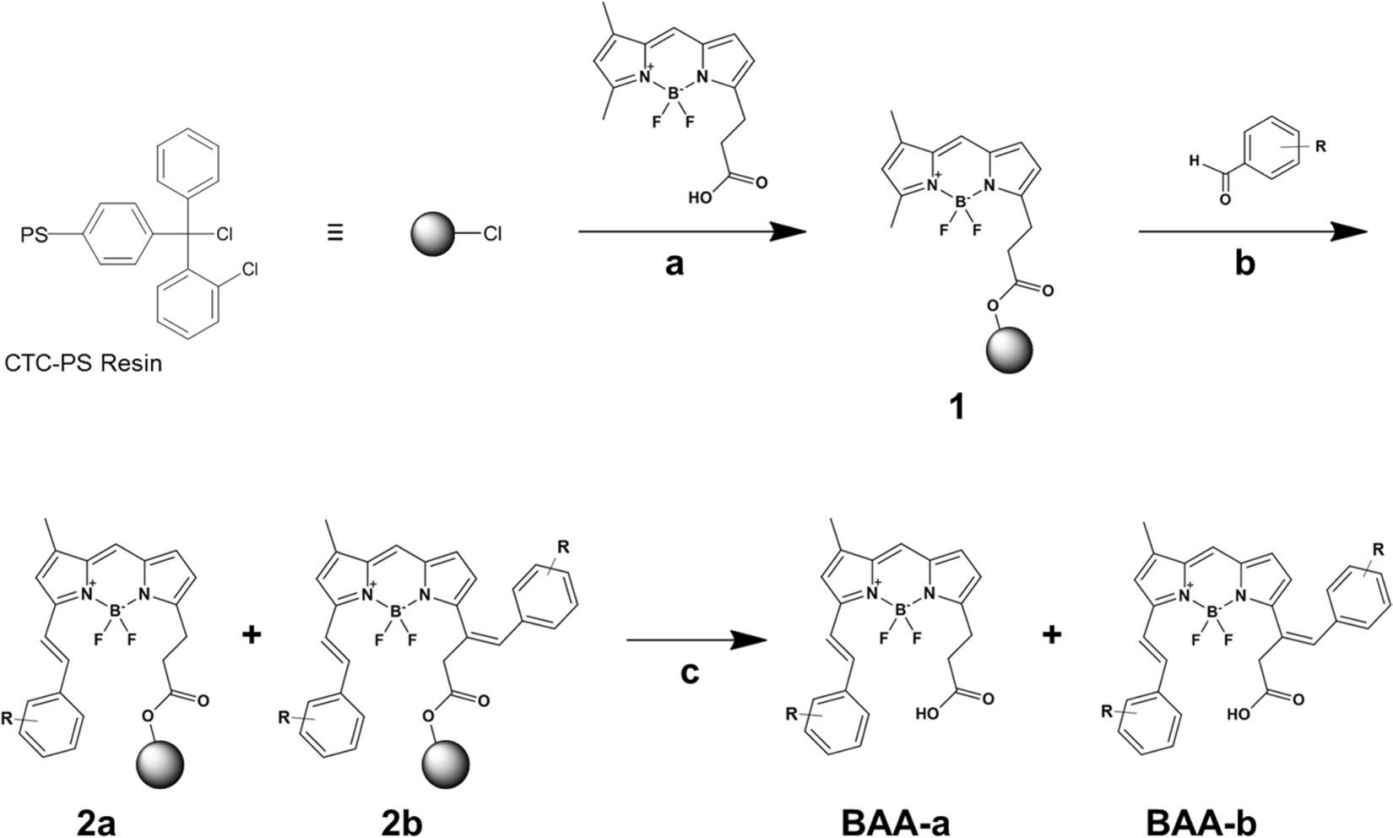
Synthesis of BAA Library. Reagents and Conditions: (a) DCM, DIEA, rt, 20 h; (b) R-CHO (79 aromatic aldehydes, see Table S1, Supporting Information), DMSO-ACN (1:1), pyrrolidine, acetic acid, 85 °C, 15 min; (c) TFA-DCM (0.5:99.5), rt, 2 × 10 min.

A library of BODIPY-based fluorophores was synthesized using solid phase synthesis to add structurally diverse styryl moieties (Fig. 1), with the goal of creating fluorophores with varied length Stokes shifts for multicolor imaging applications. Commercially available BODIPY FL was loaded onto chlorotrityl chloride polystyrene resin (CTC-PS) through its carboxylic acid moiety (**1**), where the CTC-PS acted as a protecting group for the carboxylic acid^20^. The styryl modification of BODIPY FL was carried out by reacting the core fluorophore with 79 structurally diverse aromatic aldehydes via Knoevenagel condensation (**b**)^16^. Each aromatic aldehyde reaction with BODIPY FL resulted in novel fluorophores with a single styryl modification (**BAA-a**), two styryl modifications (**BAA-b**) or a mixture of the single and double styryl addition (Table S1). The novel BAA fluorophores were cleaved from the CTC-PS resin (**c**) yielding **BAA-a**, **BAA-b** or a mixture of **BAA-a** and **BAA-b**. After purification of the cleaved material by high performance liquid chromatography (HPLC), a library of 95 novel BAA fluorophores with a minimum of 80% purity (average purity = 99%) was obtained (Table S1). 60 BAA fluorophores had one styryl modification and 35 fluorophores had two styryl modifications. NMR and MS spectra of five selected BAA fluorophores (**BAA-37a**, **BAA-22a**, **BAA-5a**, **BAA-2a** and **BAA-39a**) revealed single addition of the aromatic aldehyde occurred exclusively at the α-methyl group (Figs S1–10).

### BAA Library Photophysical and Optical Properties

**Figure 2:**
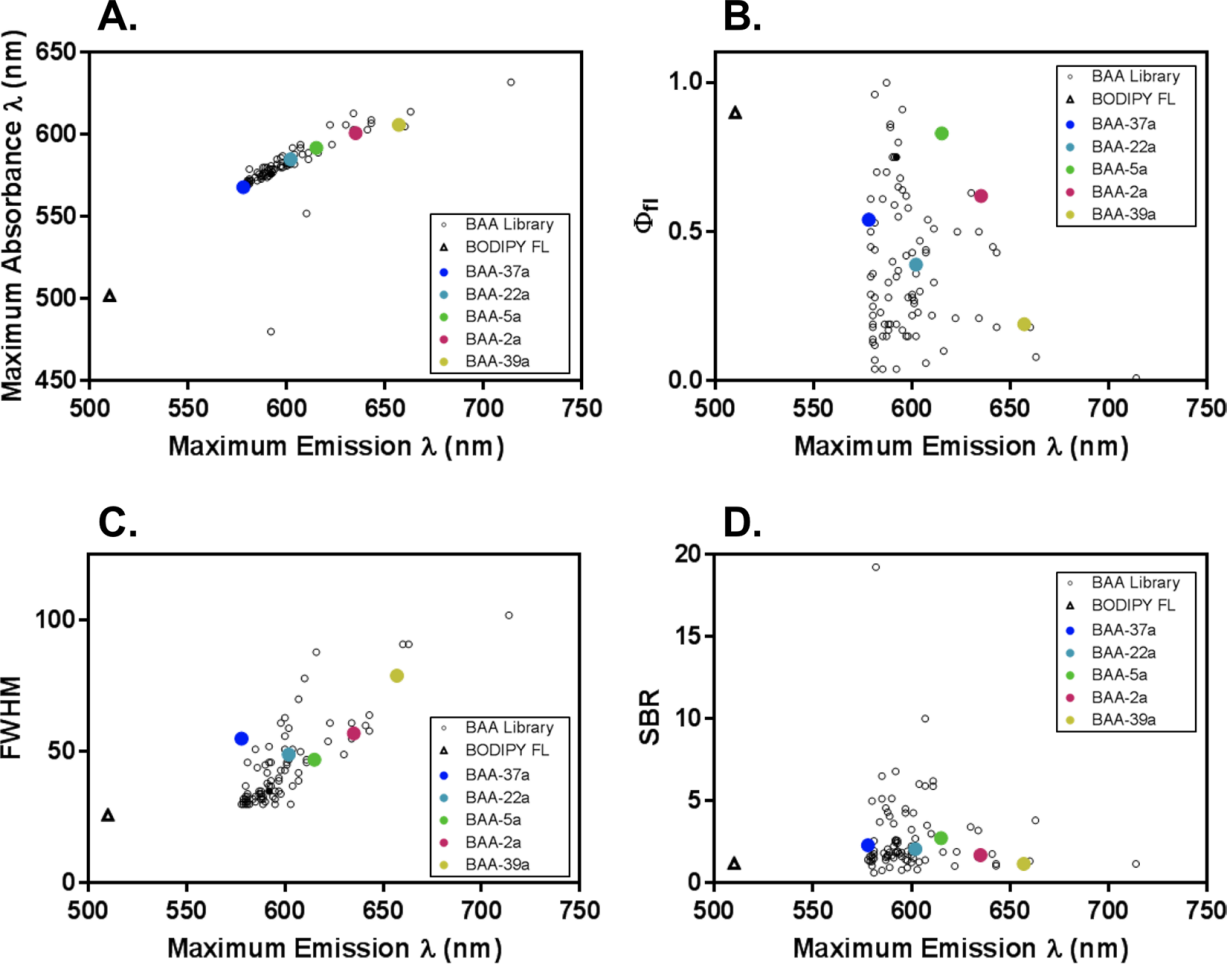
Photochemical properties & single-to-background ratio (SBR) for the BAA library. The (**A**) maximum absorbance vs. maximum emission wavelength, (**B**) quantum yield (Φ_fl_) vs. maximum emission wavelength, (**C**) full-width-at-half maximum (FWHM) vs. maximum emission wavelength, and (**D**) SBR vs. maximum emission wavelength were calculated for the BAA library and compared to BODIPY FL (triangle). The five BAA fluorophores (**BAA-37a**, **BAA-22a**, **BAA-5a**, **BAA-2a** and **BAA-39a**) selected for further study are highlighted by colored circles in each graph.

Addition of the aromatic aldehydes to the core BODIPY FL structure (λ_max ABS_ = 503 nm, λ_max EM_ = 512 nm) resulted in substantially red-shifted absorbance (ABS) and emission (EM) spectra of all novel BAA fluorophores (Fig. 2A). The BAA library ranged in maximum absorbance wavelengths from 480–614 nm and maximum emission wavelengths from 578–714 nm. Notably, 84% of the BAA fluorophores had emission wavelengths in a narrow range (578–610 nm), shifting the average emission wavelengths 75–100 nm from the original BODIPY FL compound. The BAA fluorophores ranged in Stokes shifts from 2–112 nm, where the novel fluorophores generally had longer Stokes shifts than BODIPY FL (Stokes shift = 9 nm), with an average Stokes shift of 18 nm (Table S2). The quantum yields of the BAA fluorophores varied widely, ranging from low at 0.04 to high at > 0.99, with an average of 0.38 (Fig. 2B). While the average quantum yield was lower than the original BODIPY FL (quantum yield = 0.81), it will still provide bright signal for fluorescence imaging applications. The full-width-at-half-maximum (FWHM) was quantified to determine the spectral spread in emission wavelengths for multicolor microscopy. The FWHM of the BAA library had a broad range (30–102 nm) with an average of 44 nm (Fig. 2C), which was generally wider than BODIPY FL (34 nm) as would be expected from longer Stokes shift fluorophores. The BAA fluorophores also had limited background staining in cells, which would permit high contrast when affinity conjugated for staining of specific structures (Fig. 2D).

### Organelle Specificity and SBR of BAA Library

**Figure 3:**
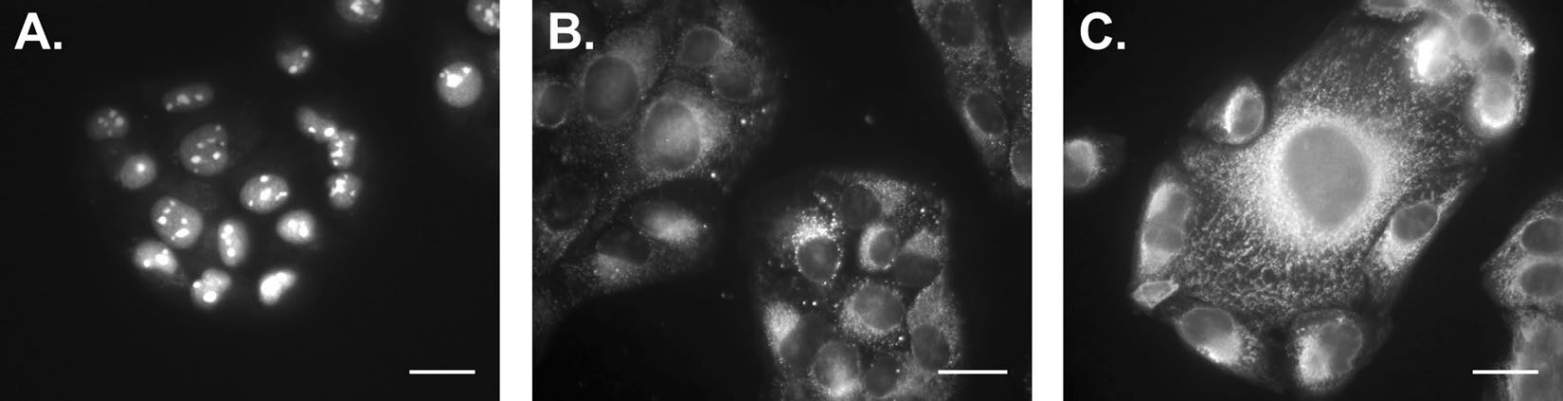
Representative images of BAA organelle specificity. The BAA fluorophores were characterized for their organelle specificity in fixed, permeabilized U2OS cells. Representative examples of (**A**) localization to nucleus and nucleolar regions by **BAA-2a**, (**B**) localization to vesicular structures by **BAA-10b** and (**C**) localization to cytosolic region by **BAA-1b** are shown. Scale bar = 20 µm.

The BAA library was screened in permeabilized, fixed U2OS cells at 10 µM to assess any organelle specificity of the novel fluorophores (Tables S3 and S4). At the selected concentration, all BAA fluorophores showed cell staining patterns with higher intensity than background autofluorescence demonstrating some level of organelle specificity. Interestingly, the cellular staining pattern varied across the library (Table S3), where three BAA fluorophores were found to localize only to the nucleus and nucleolar regions of the cell including **BAA-2a**, **BAA-30a**, and **BAA-77a** (Fig. 3A), vesicular structures were stained by 22 of the BAA fluorophores (Fig. 3B), cytosolic staining was seen from 34 of the BAA fluorophores (Fig. 3C), and the remaining 36 BAA fluorophores showed a combination of vesicular and cytosolic structural staining.

The BAA library was also screened for fluorescence staining in fixed U2OS cells at 100 nM to determine if nonspecific binding of the BAA fluorophores would be detectable at fluorophore concentrations typically used for immunofluorescence staining. The BAA fluorophores had limited staining at 100 nM concentration, where more than half of the compounds had a SBR less than 2 and ~75% had a SBR less than 3, while the average SBR for all 95 BAA compounds was 2.65 (Fig. 2D and Table S4). Background staining of 2–3 was minimal compared to the SBR seen in organelle specificity screening, where SBR ranged from 9–20, while immunofluorescence images had a minimum SBR of 10, demonstrating the utility of the BAA fluorophores for specific staining studies.

Molecular properties calculations for the BAA fluorophore library were completed where the partition coefficient (LogD), number of rotatable bonds, number of hydrogen bond donors and acceptors (HBD and HBA), polar surface area and p*K*_a_ were characterized (Table S4). Addition of the aromatic aldehydes to BODIPY FL generated a wide range of partition coefficients ranging from −4.22 to 7.12, with an average of 2.25, displaying a general increase from BODIPY FL (−2.13). The number of rotatable bonds was also highly variable in the BAA library, ranging from 5–22 with an average of 8.5, where all BAA fluorophores showed an increase of at least 3 rotatable bonds over BODIPY FL (rotatable bonds = 2) due to the increase in size of the BAA fluorophores compared to BODIPY FL. The number of hydrogen bond donors was similar across the library (1–3, with an average HBD = 1.1) and to BODIPY FL (HBD = 1), while the number of hydrogen bond acceptors showed a fairly large range from 2–12 with an average HBA = 4, differentiating the derivatives from BODIPY FL (HBA = 4) with both higher and lower values. The polar surface area of the BAA fluorophores was also widely variable (45–153, with an average of 72), where polar surface area was relatively increased in comparison to BODIPY FL (polar surface area = 45). The calculated p*K*_a_ of the carboxylic acid moiety was not substantially different across the BODIPY library ranging from 3.52–4.77 with an average of 4.21, which was also not substantially different than the p*K*_a_ of BODIPY FL (p*K*_a_ = 4.49).

### BAA Fluorophores Selected for Immunofluroescence Staining

**Figure 4:**
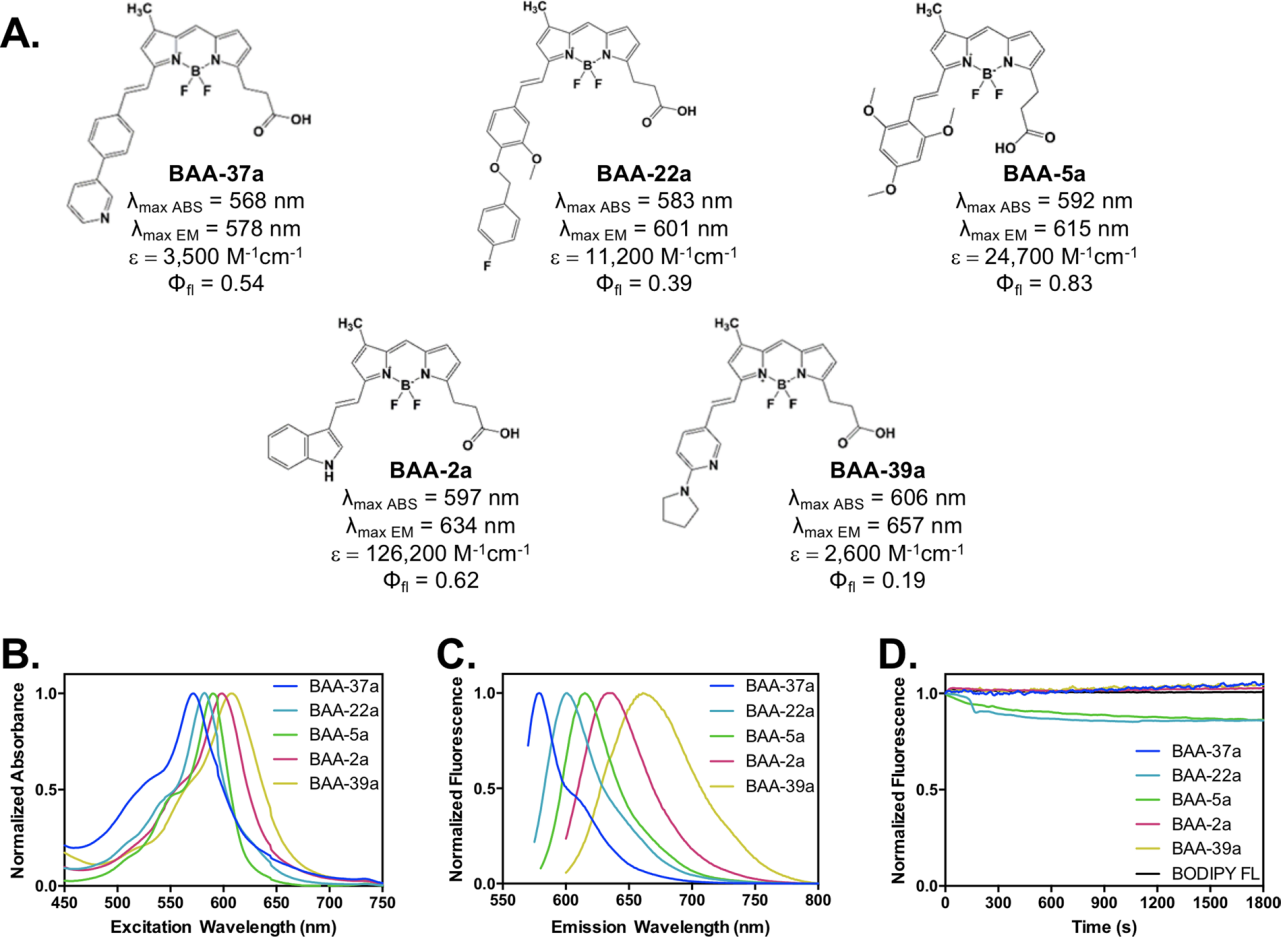
Five BAA fluorophores were selected for further characterization & multicolor imaging. **BAA-37a**, **BAA-22a**, **BAA-5a**, **BAA-2a** and **BAA-39a** were selected for further study. Their (**A**) chemical structures, absorbance (λ_max ABS_) and emission (λ_max EM_) maxima, extinction coefficient (ε) and quantum yield (Φ_fl_) are shown for all five compounds. The normalized (**B**) absorbance and (**C**) emission spectra as well as (**D**) photostability are shown for all five compounds.

Five BAA fluorophores were selected to create a multicolor immunofluorescence image with linear unmixing-based laser scanning confocal microscopy (LSM), including **BAA-37a**, **BAA-22a**, **BAA-5a**, **BAA-2a**, and **BAA-39a** (Fig. 4A). The BAA fluorophores were selected due to their relatively high quantum yield, narrow FWHM and photostability (Figs 2B,C, 4A–D) permitting five color simultaneous fluorescence microscopy, where all fluorophores could be optimally excited using a single 561 nm laser. The minimum spectral separation between the five selected BAA fluorophores was 14 nm, facilitating simultaneous spectral detection. Density functional theory (DFT) calculations were used to determine the highest occupied molecular orbital (HOMO) and lowest unoccupied molecular orbital (LUMO) energy levels of the five selected BAA fluorophores (Fig. S11). The HOMO-LUMO energy gaps ranged from 2.31–2.50 eV (Table 1). As expected, BAA fluorophores with lower energy gaps tended to have red shifted maximum absorption wavelengths.

**Table 1:** Stokes shifts and energy gaps for the five selected BAA fluorophores.

Product	λmax ABS (nm)	λmax EM (nm)	Stokes Shift (nm)	HOMO (eV)	LUMO (eV)	Energy Gap (eV)	λmax Calculated (nm)
BAA-37a	568	578	10	−5.36	−2.86	2.50	522
BAA-22a	585	602	17	−5.36	−2.90	2.46	529
BAA-5a	592	615	23	−5.04	−2.64	2.40	540
BAA-2a	597	634	37	−5.03	−2.65	2.38	543
BAA-39a	606	657	51	−5.01	−2.70	2.31	566

### Multicolor Linear Unmixing-Based Microscopy Imaging with BAA Fluorophores

Five subcellular structures in fixed U2OS cells were labeled with the selected BAA fluorophores and imaged using linear unmixing-based LSM. The carboxylic acid moiety of the BAA fluorophores was converted to an *N*-hydroxysuccinimide (NHS) ester before conjugation to the labeling proteins used to specifically target five subcellular structures with indirect immunofluorescence and lectin labeling strategies (Table 2). The inner nuclear membrane was labeled with an anti-Lamin B1 antibody via indirect immunofluorescence labeling with **BAA-37a**, the outer mitochondrial membrane was labeled with an anti-Tomm20 antibody via indirect immunofluorescence labeling with **BAA-22a**, tubulin was labeled with an anti-tubulin antibody via indirect immunofluorescence labeling with **BAA-5a**, the extracellular membrane was labeled with the lectin, wheat germ agglutinin conjugated to **BAA-2a**, and vimentin was labeled with an anti-vimentin antibody via indirect immunofluorescence labeling with **BAA-39a**.

**Table 2:** Cellular targets and protein labels used for multicolor microscopy.

Structure	Antigen Label	Secondary Antibody	BAA Fluorophore	Extinction Coefficient (M^−1^cm^−1^)	BAA:Protein Conjugation Ratio
Nuclear Membrane	Anti-lamin A (Abcam, ab8980)	goat anti-mouse IgG3 (msIgG3, 115-005-209)	BAA-37a	3,500	4.2:1
Mitochondial Membrane	Anti-TOMM20 (Abcam, ab78547)	donkey anti-rabbit IgG (rbIgG, 711-005-152)	BAA-22a	11,200	2.4:1
Tubulin	Anti-Tubulin (Millipore, MAB1864)	donkey anti-rat (ratIgG, 712-005-153)	BAA-5a	24,700	4.4:1
Extracellular Membrane	Wheat Germ Agglutinin (Vector Labs, L-102)	N/A	BAA-2a	126,200	0.2:1
Vimentin	Anti-Vimentin (Millipore, AB5733)	donkey anti-chicken (chIgG, 703-005-155)	BAA-39a	2,600	0.9:1

^*^All secondary antibodies were purchased from Jackson Immunoresearch (Jackson ImmunoResearch Laboratories, Inc., West Grove, PA).

**Figure 5:**
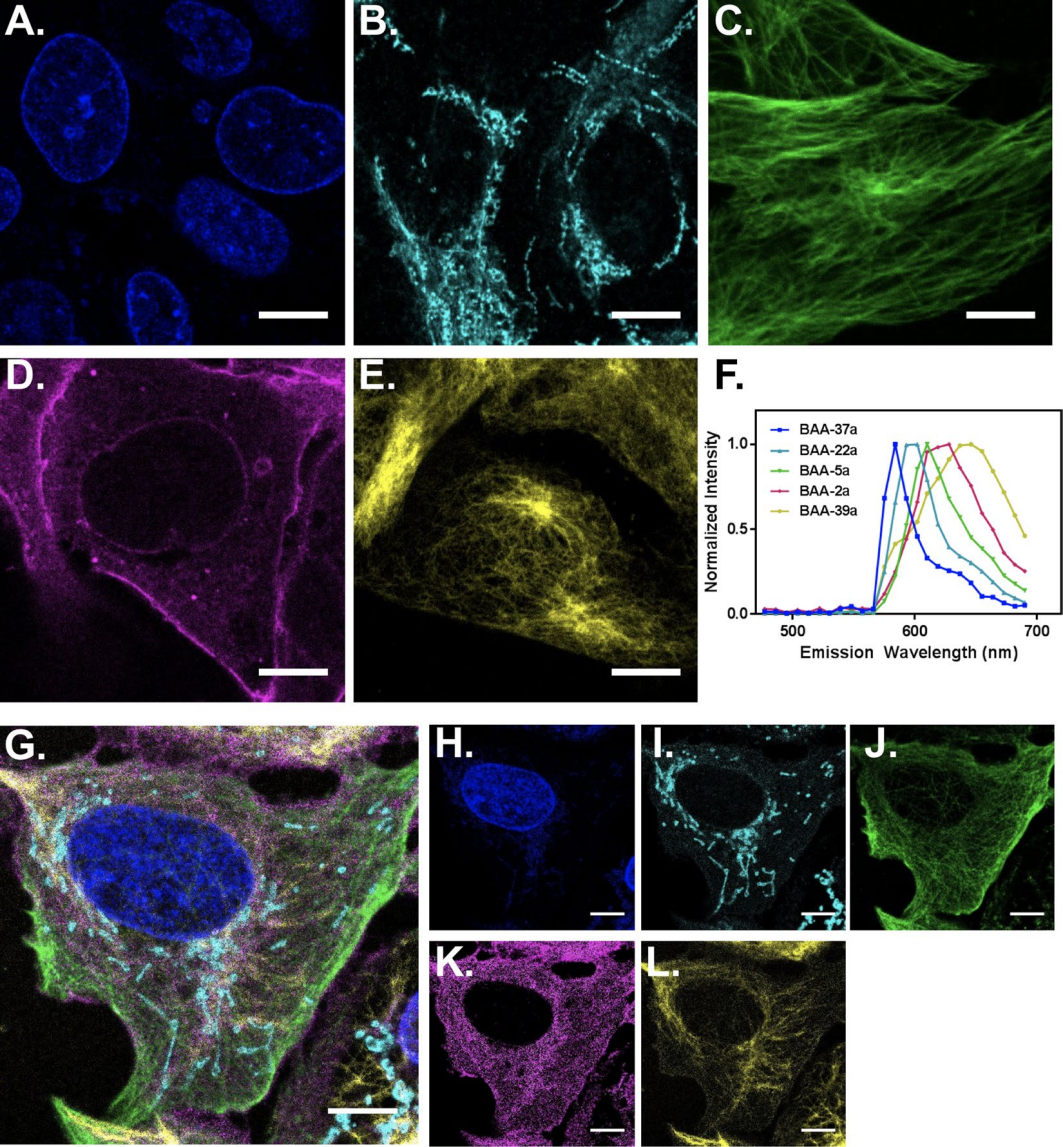
Immunofluorescence imaging of five subcellular structures. Immunofluorescence imaging using the five selected BAA fluorophores was completed on separate samples to demonstrate the structural and spectral separation of the flurophores and cellular targets. (**A**) The nuclear membrane was labeled using indirect immunofluorescence with **BAA-37a**. (**B**) The outer mitochondrial membrane was labeled using indirect immunofluorescence with **BAA-22a**. (**C**) Tubulin was labeled using indirect immunofluorescence with **BAA-5a**. (**D**) The extracellular membrane was labeled using **BAA-2a** conjugated wheat germ agglutinin. (**E**) Vimentin was labeled using indirect immunofluorescence with **BAA-39a**. (**F**) The *in situ* normalized spectral emission of each BAA fluorophore was collected using the confocal laser scanning microscopy and used for spectral unmixing in (**G**) simultaneously stained samples created using the same five selected BAA fluorophores conjugated to the validated subcellular tagging reagents. The unmixed, individual spectral channels show the (**H**) nuclear membrane, (**I**) mitochondrial membrane, (**J**) tubulin, (**K**) extracellular membrane, and (**L**) vimentin. Scale bar = 10 µm.

Each subcellular structure was labeled and imaged individually to confirm the accuracy of immunofluorescence staining (Fig. 5A–E) and to assess the *in situ* spectral emission of the five BAA fluorophores for linear unmixing of multicolor images (Fig. 5F). The *in situ* spectral emission was collected at 8.9 nm step size and was similar to the emission spectra collected in solution (Figs 2B and 5F). The only differences between the in solution and *in situ* spectra were that the emission peaks of **BAA-5a** and **BAA-39a** were blue shifted 7 and 15 nm, respectively from the in solution spectral emission. A five-color image, with all five subcellular structures labeled simultaneously, allowed for correct identification and visualization of each structure upon linear unmixing (Fig. 5G–L). Notably, labeling of tubulin (Fig. 5J) and the extracellular membrane (Fig. 5K) showed lower contrast in the multicolor image than in their corresponding individually labeled images (Fig. 5C,D). Additionally, there was minimal cross talk between the channels, determined based on the visible structures in each linearly unmixed image (Fig. 5H–L).

## Discussion

In summary, we designed and developed a library of BODIPY-based fluorophores with varied length Stokes shifts compatible with conjugation to affinity reagents for multicolor microscopy imaging applications. The diverse styryl modifications of the core BODIPY structure extended its π-conjugation resulting in substantially red-shifted absorbance and emission wavelengths^21,22^. The maximum absorbance and emission of each unique BAA fluorophore was influenced by the electron withdrawing or donating tendencies of the aromatic aldehyde used for styryl modification^23,24^.

**Figure 6:**
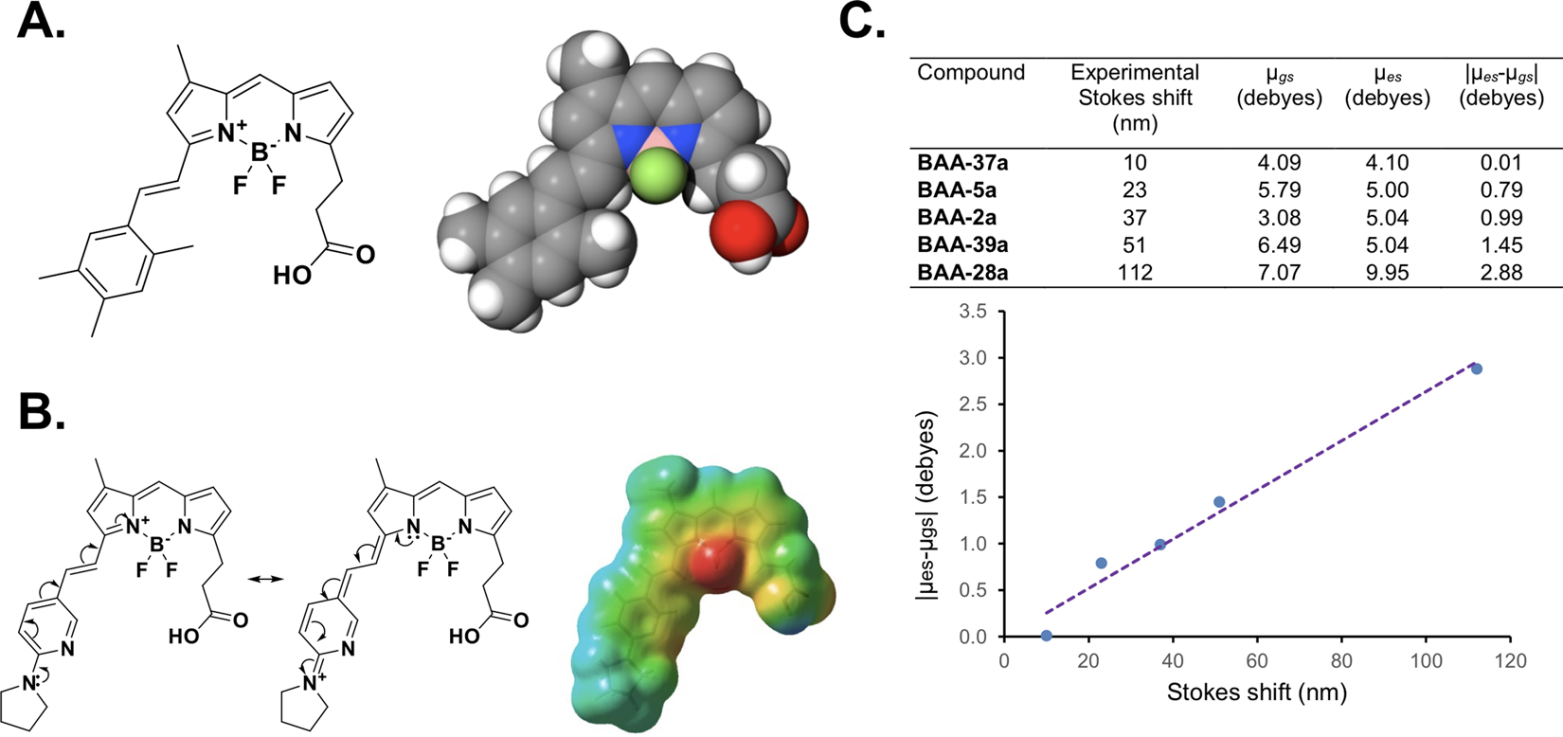
Chemical Mechanism of BODIPY-based library Stokes shift. (**A**) Compound **BAA-16a** did not have heteroatoms in its styryl extended π-system. The relatively long Stokes shift of 58 nm was attributed mainly to the bulky methyl substituents on the styryl extension of this fluorophore. (**B**) The resonance structures of **BAA-39a** and its molecular electrostatic potential surface are shown. The nitrogen in the pyrrolidine ring could share its lone pair electrons through the π-conjugated system, while the nitrogen in the pyridine ring could not. The negative charge (red) was localized between the boron and the fluorine atoms, while the positive charge (blue) was distributed along the π-conjugated system. (**C**) Correlation of the absolute value of the difference in dipole moments between the excited and ground states (|μ_*es*_-μ_*gs*_|) vs. the Stokes shift showed a linear relationship in the BAA library. The optimized geometries for the ground state (*gs*) and excited state (*es*) were obtained from DFT calculations using the B3LYP hybrid density functional with the 6–31 G(d,p) basis set.

The judicious design of steric hindrance and resonance effects in fluorophore architectures lead to enhanced Stokes shifts. For example, the relatively sterically encumbered **BAA-16a** had a Stokes shift of 58 nm without any heteroatoms in its styryl modification (Fig. 6A). All of the other fluorophores in the BAA library that exhibited Stokes shifts >30 nm possessed amine groups with lone electron pairs that delocalize into the styryl π-system. This was in agreement with a previously observed, strong effect of an electron-donating aniline on the Stokes shift of BODIPY dyes^25,26^. In the case of styryl and related BODIPYs, such as those described herein, the delocalization of the amine group lone pairs lead to charge-separated structures that were relatively strong contributors to excited state geometries. This resulted in dipole moment differences between the ground and excited states, enhancing the Stokes shifts. Compound **BAA-39a** showed two types of amine groups as part of the extended styryl framework (Fig. 6B). Only the nitrogen in the pyrrolydine ring could delocalize its lone electron pair and be part of the π-conjugated system. In contrast, the lone pair nitrogen in the pyridine ring was perpendicular to the orbitals in the aromatic system. Calculations of the molecular electrostatic potential surface showed that the positive charge was delocalized along the styryl extended π-conjugated system, while the negative charge was localized between the boron and the fluorine atoms (Fig. 6B).

DFT calculations of the dipole moment (μ) for optimized geometries in the ground and excited states for BAA compounds with varied length Stokes shifts including **BAA-2a**, **BAA-5a**, **BAA-28a**, **BAA-37a** and **BAA-39a** revealed a strong linear correlation between the absolute value of the difference in the dipole moments and Stokes shifts (Fig. 6C). These results suggest that significant changes in the geometry and charge distribution in the fluorophore excited state, can indeed produce larger Stokes shifts. Notably, the exceptional correlation between the simulations and the experimental data can potentially be used in the future design of fluorophores, where a known Stokes shift value is required, saving valuable time and synthetic resources.

Although the photophysical and optical properties varied among the novel BAA fluorophores, most compounds were compatible with excitation using a standard 561 nm laser. This enabled production of a multicolor image with a single excitation source by choosing fluorophores with different length Stokes shifts from the library including, **BAA-37a**, **BAA-22a**, **BAA-5a**, **BAA-2a**, and **BAA-39a**. Linear unmixing-based multicolor microscopy demonstrated simultaneous visualization of five subcellular structures (Figs 5G–L), facilitated by bright, spectrally distinct novel BODIPY-based fluorophores for detection and spectral separation. Tubulin labeled with **BAA-5a** and the extracellular membrane labeled with **BAA-2a**, showed lower contrast when imaged simultaneously (Figs 5J,K) compared to when imaged individually (Figs 5C,D). This was likely due to significant spectral overlap between the two fluorophores, which caused signal loss during linear unmixing (Figs 5F). Using a fluorophore with an increased red-shifted emission in place of **BAA-2a** in the five-color imaging scheme, such as **BAA-30a**, could improve multicolor imaging contrast by further increasing the spectral separation, thus improving linear unmixing.

While five fluorophores were selected for the BAA library for the multicolor microscopy imaging example demonstrated herein, additional fluorophores from the BAA library will find utility in future fluorescent imaging applications. For example, additional BAA fluorophores could be chosen to increase multiplexing capacity using a standard 561 nm laser for excitation, in addition to other common lasers such as 594 and 647 nm, where BAA fluorophores from the library showed absorbance. Likewise, BAA fluorophores could also be combined with commercially available fluorophores to create alternative color combinations and to further increase multiplexing capacity by taking advantage of both long and conventional short Stokes shift fluorophores. For example, using a combination of our five selected BODIPY fluorophores and conventional short Stokes shift fluorophores excited with 488 nm, 561 nm, 594 nm and 647 nm lasers, could result in an 8–10 color image from simultaneous fluorescence labeling.

Interestingly, we found that most BAA fluorophores had some intracellular labeling affinity, where many of our novel fluorophores labeled multiple areas of the cells, but unexpectedly some BAA fluorophores appeared to have organelle specificity (Fig. 3, Tables S3 and S4). Notably, we found three BAA fluorophores that specifically labeled the nucleus and nucleolar regions, indicating potential affinity for nucleic acids. Other BAA fluorophores appeared specific to particular vesicles, possibly localizing to the adiposomes, lysosomes or peroxisomes. Additionally, there were BAA fluorophores that appeared specific to a particular structure within the cytosol, possibly localizing to the endoplasmic reticulum, Golgi complex or mitochondria. Recent studies have shown that pH can have a strong effect on observed fluorescence signal in cellular organelles^27^. The p*K*_a_ of our novel BODIPY compounds was calculated to determine if pH could explain the organelle staining differences seen across our library, however the p*K*_a_ was not substantially different across our 95 BODIPY compounds, demonstrating the pH was not the sole reason for varied organelle accumulation. The reasons behind the precise intracellular location of the BAA fluorophores is under further investigation.

In summary, we designed, synthesized and characterized a BODIPY-based library to generate novel probes with varied length Stokes shifts for multicolor microscopy imaging applications. We found that individual BAA fluorophores have potential as organelle specific fluorophores for labeling fixed cells, but overall staining at immunofluorescence concentrations resulted in low SBR ideal for targeted imaging. We demonstrated the application of our BAA fluorophores to generate a representative five-color image over a narrow 60 nm spectral range using a single excitation wavelength. Linear unmixing-based microscopy with fluorophores in a narrow spectral window will advance multicolor imaging, where our novel probes could be combined with conventional short Stokes shift fluorophores to generate an 8–10 color multiplexed image from a simultaneously stained sample.

## Materials and Methods

### Chemicals and Fluorophores

All commercially available starting materials were used without further purification unless otherwise stated. 4,4-Difluoro-5,7-dimethyl-4-bora-3a,4a-diaza-s-indacene-3-propionic acid (BODIPY FL) was used as the starting material for all novel fluorophore synthesis (Thermo Fisher Scientific, Waltham, MA). All novel BODIPY based fluorophores were synthesized through the addition of styrl based aromatic aldehyde building blocks (Thermo Fisher Scientific or Sigma-Aldrich, St. Louis, MO). Solid phase synthesis was performed using 2-Chlorotrityl chloride polystyrene (CTC-PS) 100–200 mesh resin (EMD-Millipore, Billerica, MA). Solvents for synthesis, purification and analysis were purchased at liquid chromatography/mass spectroscopy (LCMS) purity grade (Thermo Fisher Scientific).

### Synthesis of BODIPY Aromatic Aldehyde (BAA) Fluorophore Library

The BODIPY FL was loaded onto the CTC-PS resin as follows; 500 mg of BODIPY FL in 17.2 ml of dichloromethane (DCM) was added to 5 g of CTC-PS resin in 14.9 ml of diisopropylethylamine (DIEA). The mixture was protected from light and mixed on a shaker overnight. The mixture was washed while filtering the CTC-PS resin using DCM, dimethylformamide (DMF), methanol (MeOH), DMF, and DMC, with three washes per solvent. The BODIPY FL loaded CTC-PS resin was then dried with ethyl ether, resulting in ~90% BODIPY FL loaded onto the resin, as quantified by absorbance spectroscopy and the extinction coefficient of BODIPY FL (80,000 M^−1^cm^−1^). BODIPY FL loaded CTC-PS resin (100 mg in 3 ml of 1:1 dimethylsulfoxide (DMSO):acetonitrile (ACN)) and 15 equivalents of the selected aromatic aldehyde (Table S1, 0.45 mmol in 1 ml 1:1 DMSO:ACN) were mixed with 65 equivalents of acetic acid (105 µl, 2 mmol) and 65 equivalents of pyrrolidine (150 µl, 2 mmol) at 85 °C for 15 min. The BODIPY FL loaded CTC-PS resin was washed three times each with DCM, DMF, DCM, DMF, DCM, and DMF, followed by washing three times with ethyl ether. The synthesized BODIPY aromatic aldehyde (BAA) fluorophores were then cleaved with 0.5% trifluoroacetic acid (TFA) in DCM for 10 min at room temperature. The BAA fluorophores were purified using high performance liquid chromatography (Agilent 1250 Infinity HPLC) with a C18 column (150 × 21.2 mm) using solvents A: water-formic acid (CH_2_O_2_): 99.9:0.1 and B: ACN-CH_2_O_2_: 99.9:0.1, with the gradient 90% A to 50% A (6 min) followed by 50% B to 10% B (14 min) at a flowrate of 8 ml/min. The purified samples were lyophilized for analysis (Labconco, Kansas City, MO).

### BAA Library Structural & Purity Assessment

The mass to charge (*m/z*) ratio and purity of all BAA fluorophores was assessed by tandem LCMS (Agilent 6244 time of flight LCMS with diode array detector VL+) using a C18 column (4.6 × 50 mm), where purity was determined through area under the curve (AUC) analysis of the absorbance at 254 nm (Table S1). Physiochemical properties of the BAA library (Table S4) were calculated using Marvinsketch (ChemAxon, Cambridge, MA)^28^. Density functional theory (DFT) calculations at the B3LYP/6-31 G(d,p) level were performed using Gaussian 09 (Gaussian, Inc., Wallingford, CT)^29^. ^1^H NMR spectra were recorded for the five BAA fluorophores selected for multicolor microscopy (**BAA-37a**, **BAA-22a**, **BAA-5a**, **BAA-2a** and **BAA-39a**) on (ARX-400 Avance spectrometer, Bruker, Billerica, MA). All chemical shifts (δ) reported in ppm are relative to the signals of residual solvent DMSO-d6 (2.50 ppm), and coupling constants are given in Hz. High-resolution mass spectra (HRMS) were measured for the five BAA fluorophores selected for multicolor microscopy (**BAA-37a**, **BAA-22a**, **BAA-5a**, **BAA-2a** and **BAA-39a**) on a ThermoElectron LTQ-Orbitrap high resolution mass spectrometer (Thermo Fisher) with a dedicated Accela HPLC system (Thermo Fisher).

### BAA Library Optical Property Measurements

Spectroscopic characterization was performed in DMSO in black polystyrene 96 well plates with clear bottoms (Corning, Corning, NY) using a SpectraMax M5 spectrometer with a Microplate reader (Molecular Devices, Sunnyvale, CA). Analysis included solvent corrected measurement of absorbance spectra from 400–800 nm, calculation of maximum absorbance wavelength, measurement of fluorescence emission spectra using the maximum absorbance for excitation from 10 nm above the excitation to 800 nm, calculation of the maximum fluorescence emission wavelength, measurement of quantum yield, and calculation of the full-width-at half maximum (FWHM) (Table S2). Quantum yields (Φ_fl_) were calculated by comparing the area under the emission spectrum of the BAA fluorophore to a reference fluorophore solution of Fluorescein, Rhodamine B, or Cresyl violet, at three concentrations using equation (1), where *Grad* represents the gradient from the plots of integrated fluorescence intensity vs. absorbance at three concentrations and ɳ is the refractive index of the solvent^30^. Fluorescein in 0.1 M sodium hydroxide (NaOH) (Φ_fl_ = 0.91)^31^ was used as the reference for all BAA fluorophores with a maximum absorbance of 480–510 nm. For quantum yield measurements using Fluorescein, excitation at 470 nm was used with the emission spectra integrated from 490–800 nm. Rhodamine B in ethanol (Φ_fl_ = 0.70)^32,33^ was used as the reference for all BAA fluorophores with a maximum absorbance of 511–595 nm. For quantum yield measurements using Rhodamine B, excitation at 525 nm was used with the emission spectra integrated from 545–800 nm. Cresyl violet in methanol (Φ_fl_ = 0.54)^32^ was used as the reference for BAA fluorophores with a maximum absorbance above 595 nm. For quantum yield measurements using Cresyl violet, an excitation at 570 nm was used with the emission spectra integrated from 590–800 nm.

1 $${\Phi }_{fl}^{sample}={\Phi }_{fl}^{reference}(\frac{Gra{d}^{sample}}{Gra{d}^{reference}}){(\frac{{{\rm{\eta }}}^{sample}}{{{\rm{\eta }}}^{reference}})}^{2}$$

### BAA Photostability Measurements for Select BAA Fluorophores

Photostablity of the five BAA fluorophores selected for multicolor microscopy (**BAA-37a**, **BAA-22a**, **BAA-5a**, **BAA-2a** and **BAA-39a**) and the parent BODIPY FL was measured in a 9:1 mixture of phosphate buffered saline (PBS)/DMSO. Fluorescence emission measurements were collected using a quartz cuvette in a SpectraMax M5 spectrometer (Molecular Devices). Fluorescence emission was monitored at the emission maximum of each compound every 5 seconds over a 30 minute period and reported as the normalized fluorescence intensity.

### Cell Culture

The U2OS osteosarcoma human cell line was cultured in phenol red free Dulbecco’s Modified Eagle Medium (Thermo Fisher Scientific) supplemented with 10% fetal bovine serum (VWR, Radnor, PA) and 1% Penicillin-Streptomycin-Glutamine (Thermo Fisher Scientific) at 37 °C and 5% CO_2_. Cells were plated in a 96-well glass bottom plates (Cellvis, Mountain View, CA) and incubated for 3 days to reach ~50% confluency prior to staining and imaging studies.

### BAA Library Staining for Organelle Specificity & SBR Calculations

Cells were pre-extracted with 0.5% Triton X-100 in 1× phosphate buffered saline (PBS) for 20 s, fixed with 0.4% glutaraldehyde (GA, Electron Microscopy Science, Hatfield, PA) and 0.25% Triton X-100 in PBS for 90 s, washed again with PBS and finally fixed with 3% GA in PBS for 15 min. Cells were washed with PBS (3 × 5 min), reduced with 10 mM sodium borohydride for 10 min, washed again with PBS (3 × 5 min), and blocked with 5% bovine serum albumin (BSA) in PBS for 10 min. BAA fluorophores were diluted to 10 µM in PBS for organelle screening and 100 nM for SBR screening. The diluted BAA fluorophores were incubated with the fixed, permeabilized cells for 30 min after which the fluorophore was removed and the cells were washed with PBS (3 × 5 min). Cells were counterstained with 4′,6-Diamidine-2′-phenylindole dihydrochloride (DAPI) at 1 µg/ml for 5 min and washed with PBS (3 × 5 min) prior to imaging for organelle specificity and SBR measurements.

Cell imaging was completed using a Zeiss AxioObserver (Carl Zeiss Microscopy GmbH, Jena, Germany), with a PhotoFluor II (89 North, Burlington, VT) excitation source and 63 × oil immersion objective. Samples were imaged with the optimal bandpass excitation and emission filter sets (Chroma Technology, Bellows Falls, VT) based on the BAA optical property measurements, where one of three filter sets was used including (1) 470 ± 20 nm excitation and 525 ± 25 nm emission, (2) 545 ± 12.5 nm excitation and 605 ± 35 nm emission, or (3) 620 ± 30 nm excitation and 700 ± 37.5 nm emission. Organelle specificity images were collected at exposure times ranging from 1.5–10 sec. Control images of unlabeled cells were collected at the same exposure times to confirm fluorescence was from BAA fluorophore staining and not from autofluorescence alone. Images for SBR analysis were collected at 1.5, 5, and 10 sec exposure times. Two images were obtained for each exposure time, one image to capture the DAPI using 405 ± 20 nm excitation and 470 ± 20 nm emission, and a second image to capture the BAA fluorophore using its appropriate filter set. Different regions of interest were imaged for each exposure time to ensure photobleaching was not affecting SBR measurements.

Image analysis was performed using QiTissue software (Quantitative Imaging Systems, Pittsburgh, PA). All images were first corrected for camera or scanning sensor bias, as well as dark current introduced at longer exposure times. These values were calculated and subtracted to allow proper computation of the ratios described below. The DAPI images enabled automatic detection of cell nuclei and an estimated location of the surrounding cytoplasm. Regular thresholding would introduce a measurement bias in situations with varied staining results. Instead, non-linear preprocessing, and a-priori knowledge based detection was used to define the organelles, regardless of their variation in intensities. This resulted in a reliable estimate of the area occupied by the target of labeling protocol for each type of image. The calculation then proceeded to determine the average intensity of area occupied (I_o_) and the average intensity of the background (I_b_) for each sample, and baseline intensity for each filter set *f* with no sample present (I_z*f*_). The SBR at each exposure time was calculated using equation 2. The final SBR reported was calculated as the average (avg) SBR measured across the three exposure times for each sample.

2 $$SBR=(\frac{Io-Izf}{avg(Ibf)-Izf})$$

### Protein & Antibody Labeling with BAA Fluorophores

BAA fluorophores **BAA-2a**, **BAA-5a**, **BAA-22a**, **BAA-37a**, and **BAA-39a** were selected for multicolor cell imaging studies based on their optical property measurement. These five BAA fluorophores were converted from their carboxylate form to an amine-reactive NHS ester prior to protein conjugation. BAA fluorophores were diluted into DMF with 2.2 equivalents of 1-[Bis(dimethylamino)methylene]-1H-1,2,3-triazolo[4,5-b]pyridinium 3-oxid hexafluorophosphate (HATU) and 5.8 equivalents of DIEA and mixed for 30 min. Four equivalents of *N*-hydroxysulfosuccinimide (sulfo-NHS) were added and mixed for an additional 60 min. The reaction was diluted with ethyl acetate (2 ml) and washed with water (500 µl) to separate the NHS ester functionalized BAA fluorophore from any remaining reactants. The NHS ester functionalized BAA fluorophore in ethyl acetate was dried by lyophilization. LCMS analysis was used to confirm >90% NHS conversion for each BAA fluorophore.

Five targets were selected for multicolor microscopy and labeled using BAA fluorophores conjugated to the appropriate affinity proteins (Table 2). Conjugates were made using standard NHS ester reaction conditions, where the protein or antibody was buffered exchanged into 1× PBS, pH adjusted to 8.3 with 1 M sodium hydroxide, and mixed with the NHS ester functionalized BAA fluorophore in a 3:1 fluorophore to affinity tag molar ratio. The mixture was rocked gently at room temperature protected from light for 3 hours. The conjugation reactions were purified using fast protein liquid chromatography (FPLC, NGC Quest 10 Plus Chromatography System, Bio-Rad, Hercules, CA) by size exclusion (P6 gel filtration column, 40 × 12.6 mm, Bio-Rad). Final fluorophore concentration, protein concentration and conjugation ratios were determined using absorbance spectroscopy (Table 2).

### Immunofluorescence Staining of Cellular Structures

Live cells were incubated with **BAA-2a** conjugated to WGA to label the cell membrane at 20 µg/ml WGA in PBS for ~10 s before adding paraformaldehyde (PFA, Electron Microscopy Science) to a final concentration of 2% PFA and incubating for 10 min at 37 °C and 5% CO_2._ The media and fixative mixture was exchanged with fresh 2% PFA and incubated for an additional 20 min at 37 °C and 5% CO_2_. Cells were pre-extracted with 0.5% Triton X-100 in PBS for 20 s, fixed with 0.4% GA and 0.25% Triton X-100 in PBS for 90 s, washed with PBS and finally fixed with 3% GA in PBS for 15 min. Cells were then incubated with primary antibodies in 5% BSA overnight at 4 °C, protected from light. The primary antibodies were incubated at 10 μg/ml anti-lamin A, 10 μg/ml anti-TOMM20, 10 μg/ml anti-tubulin, and 6.6 μg/ml anti-vimentin. Following overnight incubation, the cells were washed with PBS (3 × 5 min) then incubated with the fluorophore conjugated secondary antibodies at 30 μg/ml **BAA-37a**-msIgG3, 2 μg/ml **BAA-22a**-rbIgG, 20 μg/ml **BAA-5a**-ratIgG, and 50 μg/ml **BAA-39a**-chIgG as measured by protein concentration in 5% BSA for 30 min at room temperature protected from light. The antibody stained cells were then washed with PBS (3 × 5 min) and post-fixed with 4% PFA in PBS for 10 min before a final PBS wash. For images with single BAA fluorophore labeling, cells without **BAA-2a**-WGA were used.

### Linear Unmixing-Based Confocal Laser Scanning Microscopy

Images were acquired using a Zeiss LSM 880 with Airyscan using a plan-apochromatic 63 × (NA = 1.4) oil immersion objective. Images were collected in lambda mode with the following settings: laser 561 nm (DPSS 561-10), 0.05%; beam splitter: MBS 458/561; filter: 410–695 nm; pixel dwell: 48.8 µs, average: line 1; master gain: 900; pinhole size: 60 um; and acquisition area: 1384 × 1384 pixels, 16 bit. Spectral data were acquired from 410–695 nm using samples labeled with individual BAA fluorophores as well as samples labeled with all five BAA fluorophore simultaneously. Zen imaging software (Zeiss) was used for spectral unmixing and FIJI ImageJ was used to process the final images^34^.

## Electronic supplementary material

Supplementary Information
