# Supplementary material for: Varied Length Stokes Shift BODIPY-Based Fluorophores for Multicolor Microscopy
Source: Sci Rep. 2018 Mar 15;8:4590. doi: 10.1038/s41598-018-22892-8 (PMC5854673; doi:10.1038/s41598-018-22892-8)
Supplement: Supplementary file 1 — Supplementary Information [file 41598_2018_22892_MOESM1_ESM.pdf]

Supplementary Information

Varied Length Stokes Shift BODIPY-Based Fluorophores for Multicolor Microscopy

Amy M. Bittel[1], Ashley M. Davis[1], Lei Wang[1], Michel A. Nederlof[4], Jorge O. Escobedo[5], Robert M. Strongin[5], Summer L. Gibbs[1,2,3§]

[1]Biomedical Engineering Department, [2]Knight Cancer Institute, [3]OHSU Center for Spatial Systems Biomedicine, Oregon Health & Science University, Portland, OR 97201, [4]Quantitative Imaging, Pittsburgh, PA 15238, [5]Department of Chemistry, Portland State University, Portland, OR 97201

[§]Corresponding Author:

Summer L. Gibbs, Ph.D.
Oregon Health & Science University
Collaborative Life Sciences Building
2730 SW Moody Ave, Mail Code: CL3SG
Portland, OR 97201
Email: gibbss@ohsu.edu
Phone: 503-494-8940

Table S1: *Chemical structures and LCMS calculated purity data for BAA library.*

Product	Aromatic Aldehyde	Final Structure	m/z calc	m/z exp	RT (min)	Purity (%)
BAA-1b	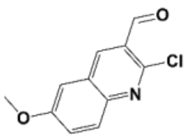	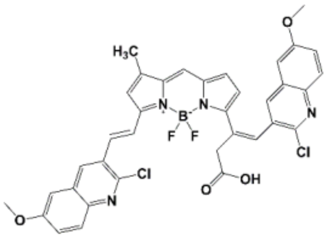	698.15	699.15	7.83	92
BAA-2a	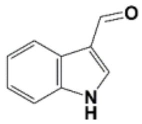	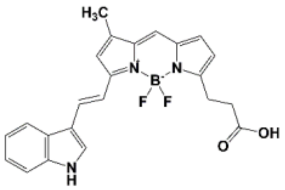	419.16	400.16[a]	7.66	>99
BAA-3a	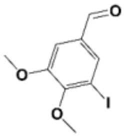	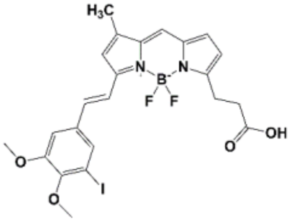	566.07	567.95	8.07	98
BAA-3b	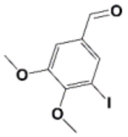	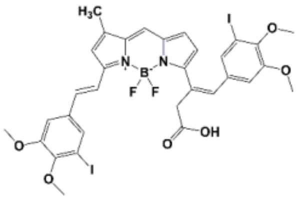	840.02	863.25[b]	8.21	97
BAA-4a	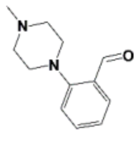	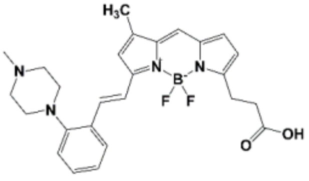	478.24	479.24	6.02	>99
BAA-5a	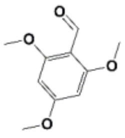	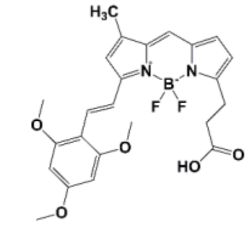	470.18	471.19	8.26	>99

Product	Aromatic Aldehyde	Final Structure	m/z calc	m/z exp	RT (min)	Purity (%)
BAA-6a	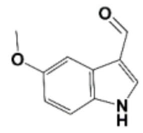	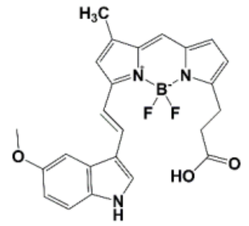	449.17	472.18[b]	7.69	82
BAA-7a	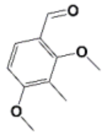	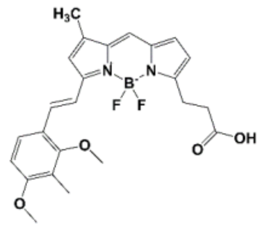	454.19	477.27[b]	8.64	83
BAA-7b	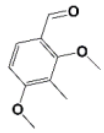	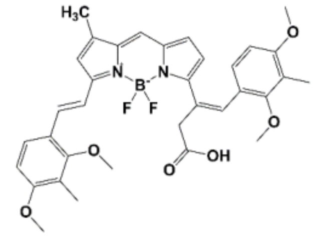	616.26	617.26	7.32	88
BAA-8a	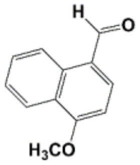	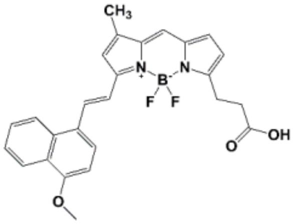	460.18	461.19	8.31	80
BAA-9a	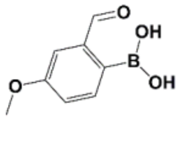	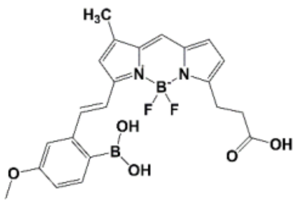	454.17	455.13	8.15	92
BAA-10b	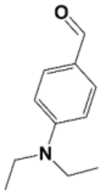	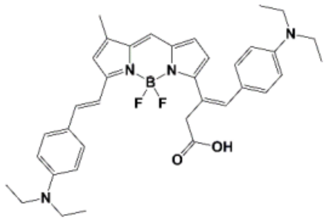	610.33	611.35	9.45	>99
BAA-11a	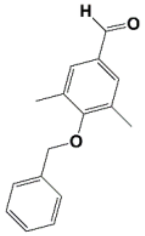	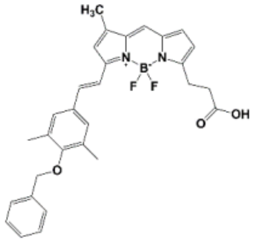	514.22	515.22	8.98	91

Product	Aromatic Aldehyde	Final Structure	m/z calc	m/z exp	RT (min)	Purity (%)
BAA-12a	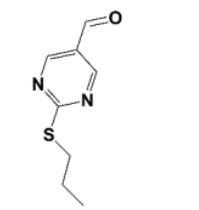	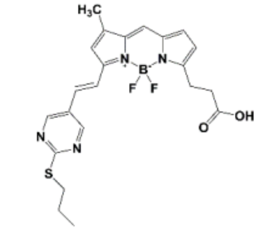	456.16	474.19[c]	7.69	88
BAA-13a	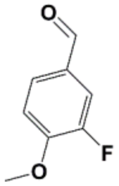	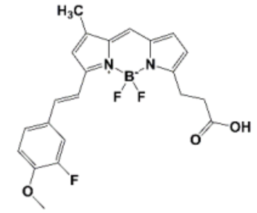	428.15	429.15	7.28	90
BAA-14a	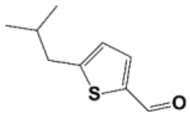	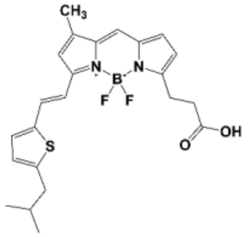	442.17	465.16[b]	9.153	83
BAA-15a	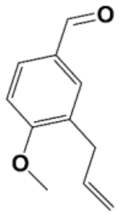	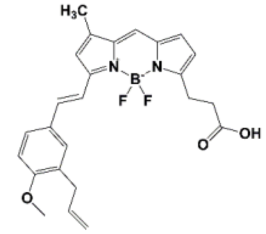	450.19	451.19	8.83	96
BAA-16a	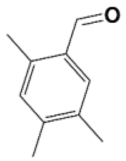	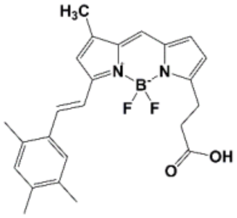	422.2	445.19[b]	9.08	98
BAA-17a	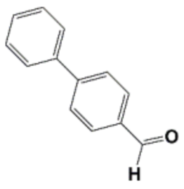	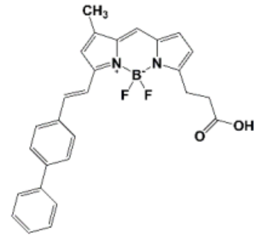	456.18	479.17[b]	9.09	85
BAA-18a	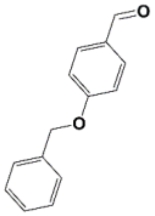	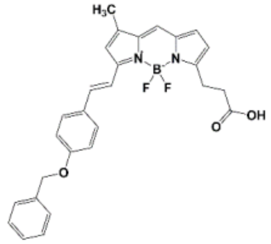	486.19	509.18[b]	8.94	92

Product	Aromatic Aldehyde	Final Structure	m/z calc	m/z exp	RT (min)	Purity (%)
BAA-19a	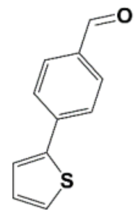	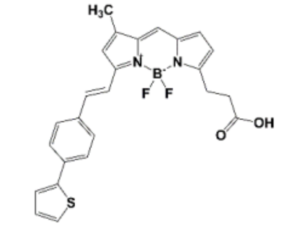	462.14	485.13[b]	8.94	96
BAA-20a	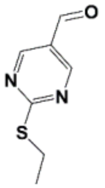	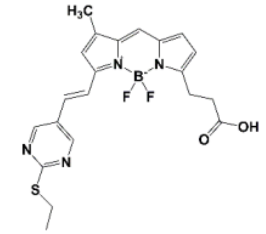	442.14	443.15	8.07	98
BAA-21a	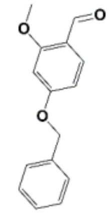	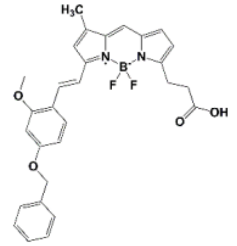	516.2	497.21[a]	9.13	95
BAA-21b	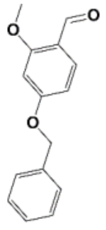	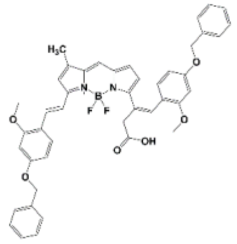	740.29	741.29	7.82	92
BAA-22a	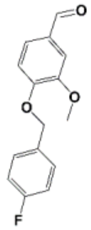	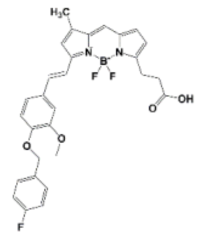	534.19	515.20[a]	8.65	94
BAA-22b	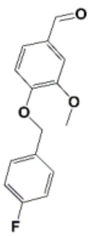	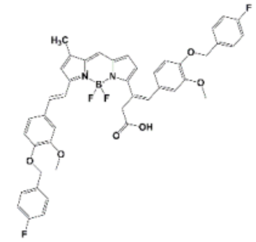	776.27	777.28	7.51	90
BAA-23a	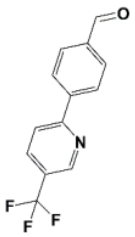	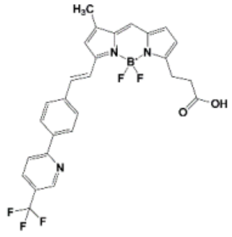	525.16	526.18	9.05	>99

Product	Aromatic Aldehyde	Final Structure	m/z calc	m/z exp	RT (min)	Purity (%)
BAA-23b	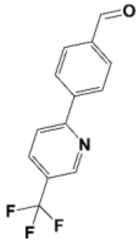	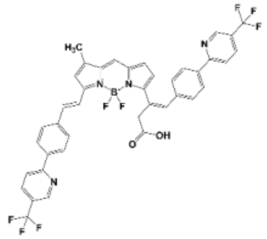	758.21	739.13[a]	7.93	89
BAA-24b	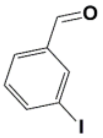	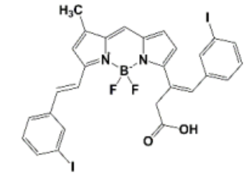	719.98	700.98[a]	7.46	94
BAA-25a	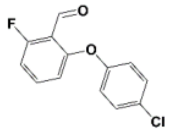	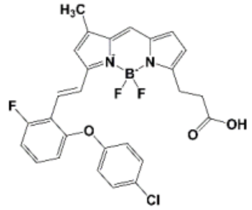	524.73	547.12[b]	9.30	93
BAA-25b	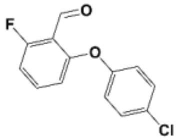	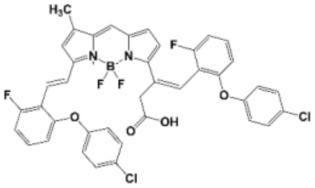	756.14	757.15	7.88	95
BAA-26b	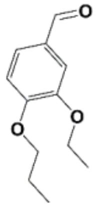	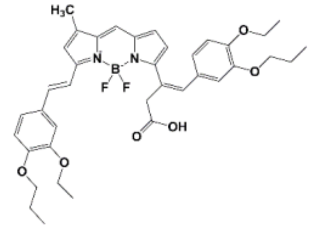	672.32	673.33	7.56	98
BAA-27b	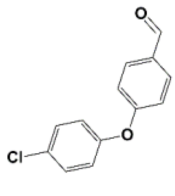	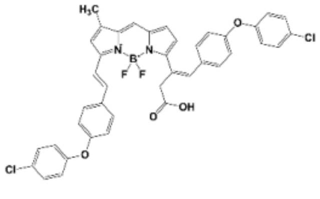	720.16	683.22[d]	7.19	97
BAA-28a	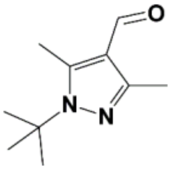	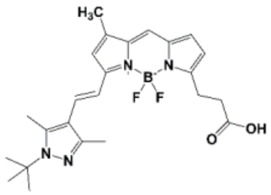	454.24	435.28[a]	6.69	88

Product	Aromatic Aldehyde	Final Structure	m/z calc	m/z exp	RT (min)	Purity (%)
BAA-28b	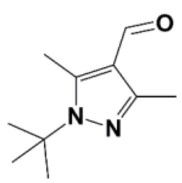	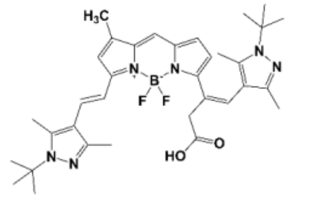	616.35	617.37	6.99	97
BAA-29b	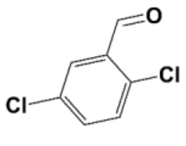	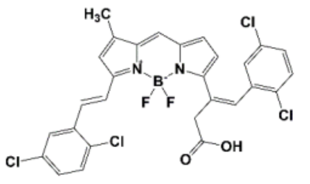	604.03	605.03	8.14	86
BAA-30a	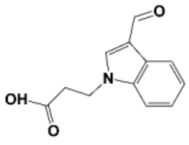	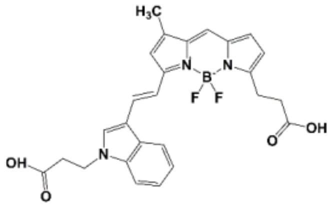	491.18	492.19	7.41	96
BAA-31b	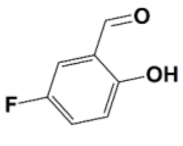	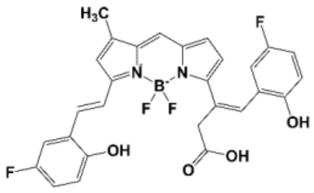	536.28	497.15[d]	6.34	94
BAA-32a	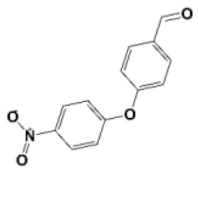	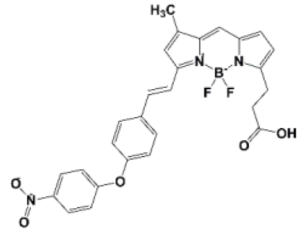	517.16	478.15[d]	8.77	>99
BAA-33a	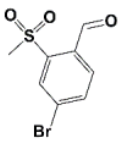	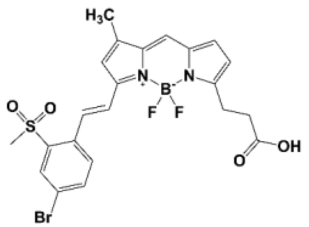	536.04	559.02[b]	7.94	98
BAA-34b	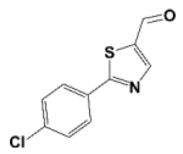	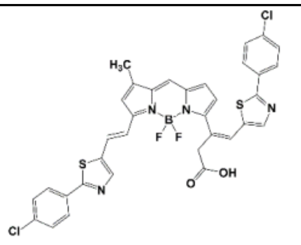	702.07	683.06[a]	7.69	94

Product	Aromatic Aldehyde	Final Structure	m/z calc	m/z exp	RT (min)	Purity (%)
BAA-35b	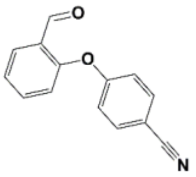	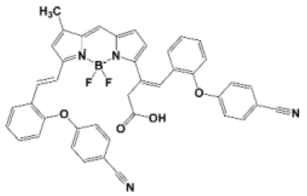	702.22	703.15	8.49	86
BAA-36b	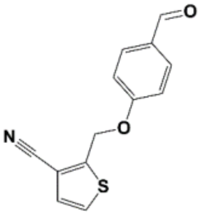	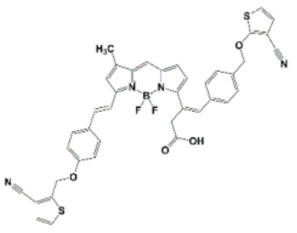	742.17	743.18	8.16	94
BAA-37a	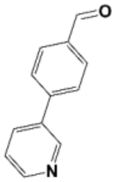	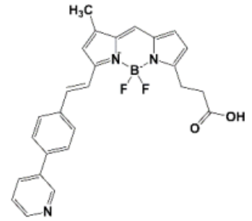	457.18	438.18[a]	5.75	88
BAA-37b	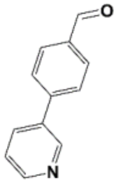	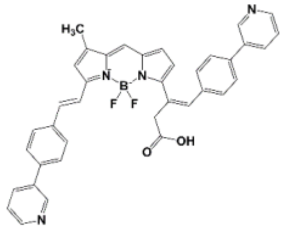	622.24	623.24	5.78	>99
BAA-38a	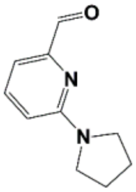	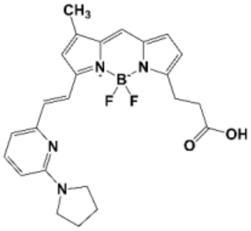	450.2	451.21	6.28	99
BAA-39a[g]	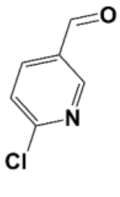	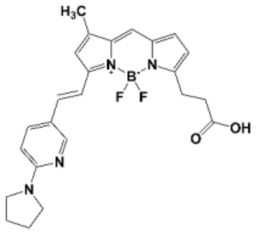	415.11	451.21[g]	5.99	94
BAA-40b	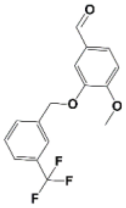	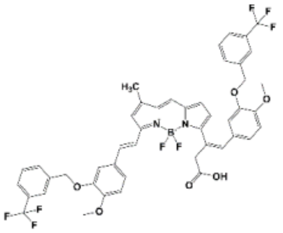	876.26	877.27	7.96	89

Product	Aromatic Aldehyde	Final Structure	m/z calc	m/z exp	RT (min)	Purity (%)
BAA-41b	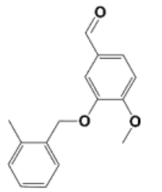	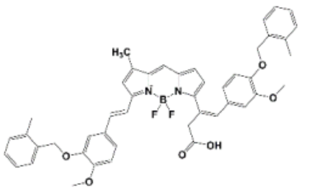	768.32	769.33	7.61	94
BAA-42a	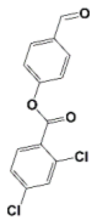	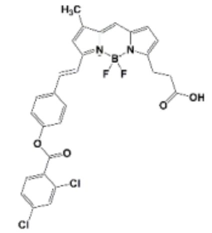	568.09	501.17[e]	6.17	82
BAA-43a	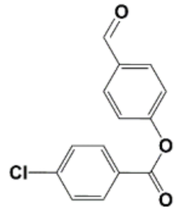	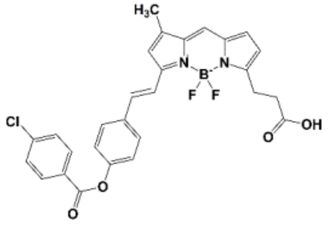	534.13	501.17[f]	6.13	94
BAA-44b	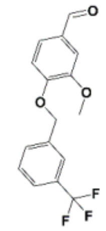	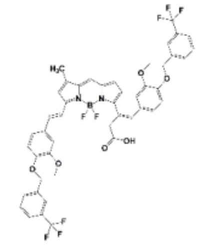	876.26	877.27	8.15	92
BAA-45a	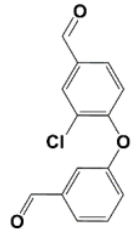	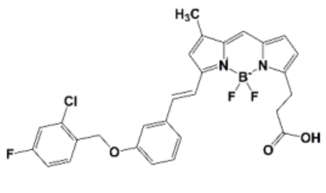	538.75	562.14[b]	9.29	>99
BAA-45b	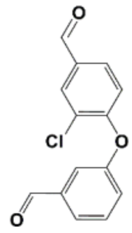	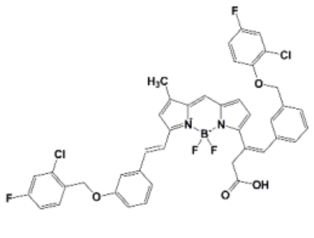	784.17	785.17	8.24	96
BAA-46b	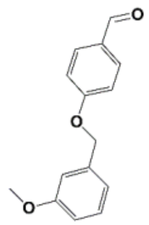	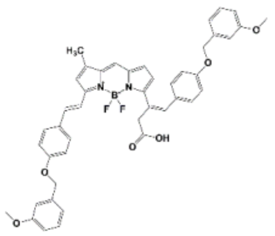	740.29	741.28	7.69	94

Product	Aromatic Aldehyde	Final Structure	m/z calc	m/z exp	RT (min)	Purity (%)
BAA-47a	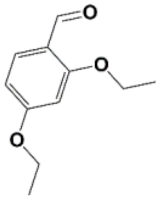	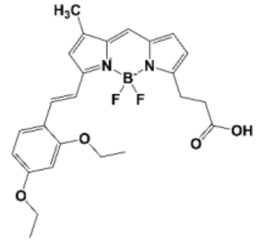	468.2	491.20[b]	8.62	94
BAA-48a	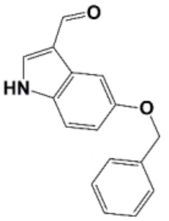	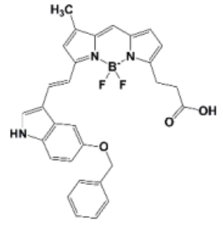	525.2	548.19[b]	8.24	>99
BAA-49b	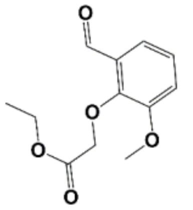	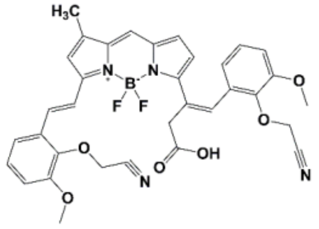	638.21	619.21[a]	6.58	>99
BAA-50a	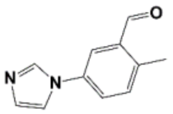	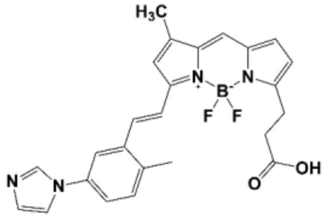	460.19	483.18[b]	8.07	>99
BAA-50b	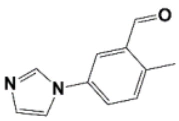	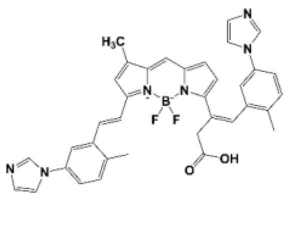	628.26	629.25	6.94	>99
BAA-51a	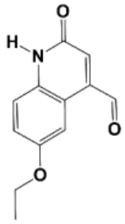	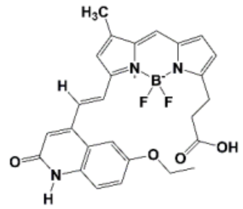	491.18	514.16[b]	7.59	89
BAA-52a	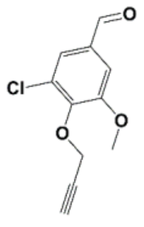	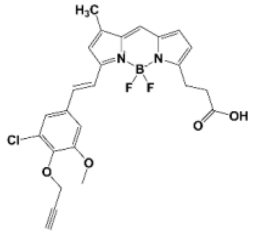	498.13	499.12	7.86	83

Product	Aromatic Aldehyde	Final Structure	m/z calc	m/z exp	RT (min)	Purity (%)
BAA-53a	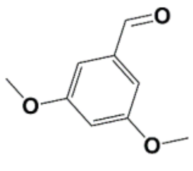	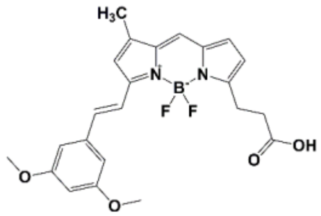	440.17	441.17	7.57	>99
BAA-54a	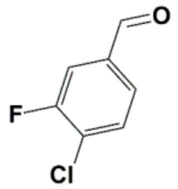	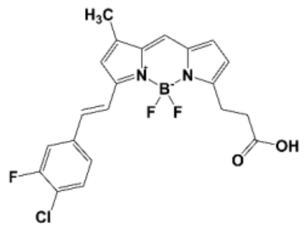	432.1	433.09	7.98	89
BAA-55a	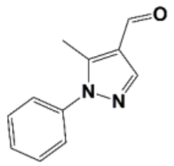	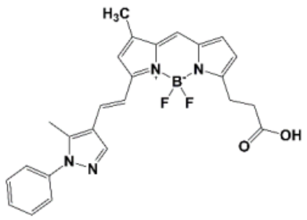	460.19	461.18	7.30	>99
BAA-55b	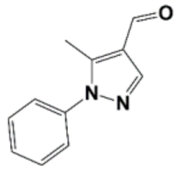	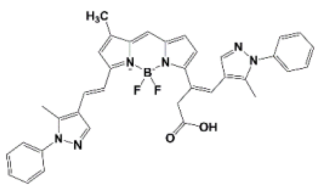	628.26	629.26	6.89	89
BAA-56a	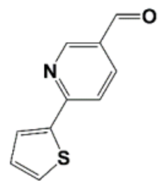	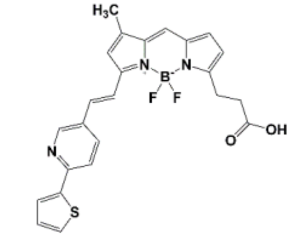	463.13	464.13	5.87	>99
BAA-56b	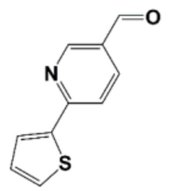	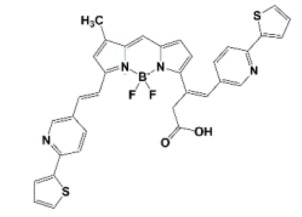	634.15	635.15	7.84	84
BAA-57a	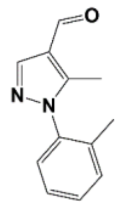	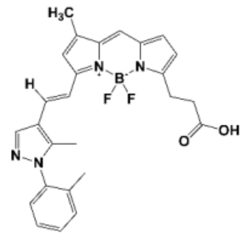	474.2	475.21	6.10	85

Product	Aromatic Aldehyde	Final Structure	m/z calc	m/z exp	RT (min)	Purity (%)
BAA-57b	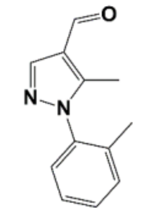	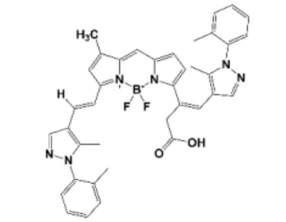	656.29	657.30	8.01	>99
BAA-58a	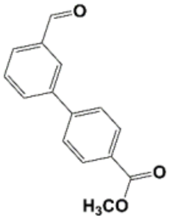	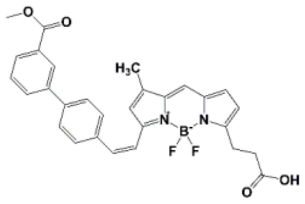	514.19	515.18	8.20	>99
BAA-59a	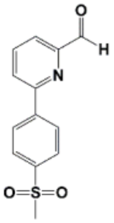	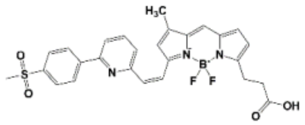	535.15	553.16[c]	7.37	96
BAA-60b	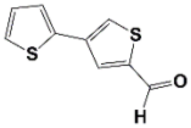	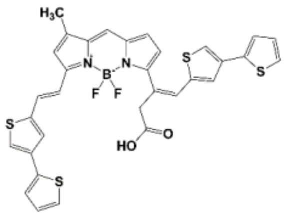	644.07	645.08	8.11	99
BAA-61a	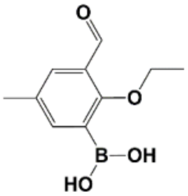	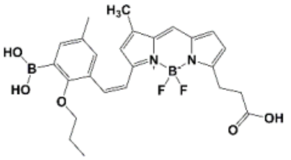	496.22	497.21	7.32	98
BAA-62a	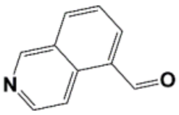	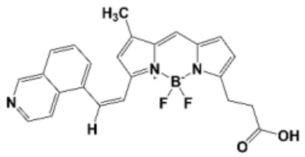	431.16	449.16[c]	6.81	>99
BAA-63a	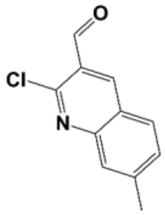	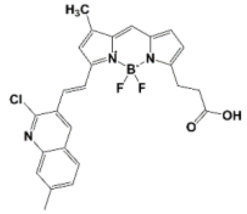	479.14	502.15[b]	7.05	93

Product	Aromatic Aldehyde	Final Structure	m/z calc	m/z exp	RT (min)	Purity (%)
BAA-64a	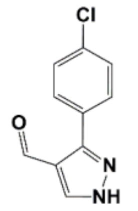	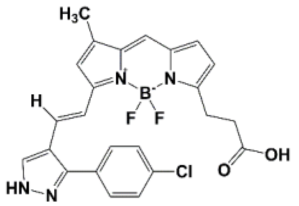	480.13	481.11	7.50	91
BAA-65a	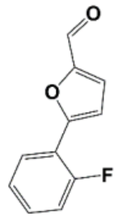	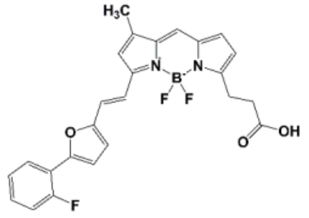	464.15	427.14[d]	8.82	88
BAA-66a	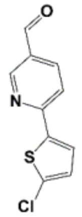	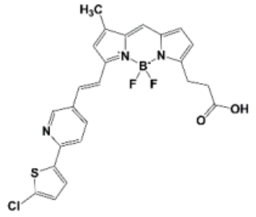	497.09	498.10	9.09	93
BAA-67a	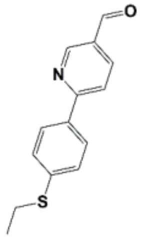	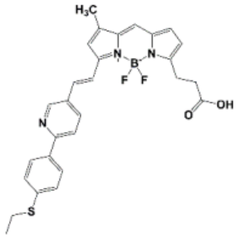	517.18	518.18	9.02	92
BAA-68a	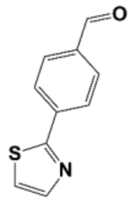	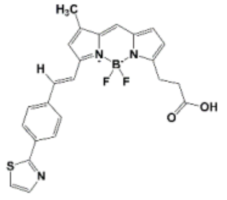	463.13	464.14	8.27	>99
BAA-69a	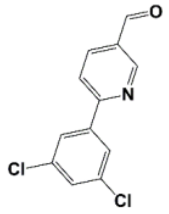	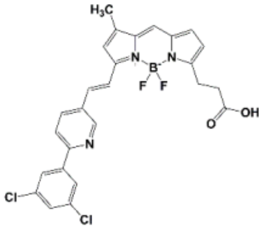	525.1	526.09	9.08	>99
BAA-70a	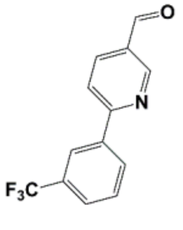	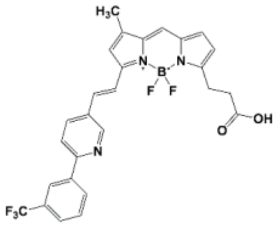	525.16	526.18	8.95	99

Product	Aromatic Aldehyde	Final Structure	m/z calc	m/z exp	RT (min)	Purity (%)
BAA-71a	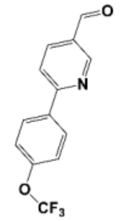	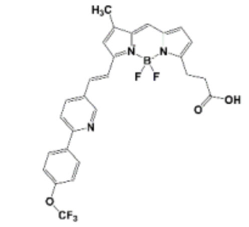	541.16	542.17	9.06	>99
BAA-72a	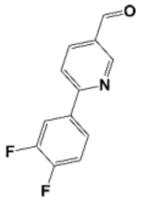	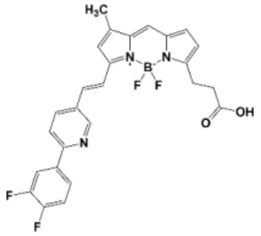	493.16	511.17[c]	8.13	98
BAA-73a	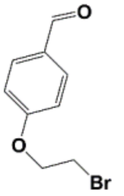	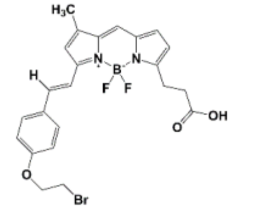	502.09	503.08	7.95	>99
BAA-74a	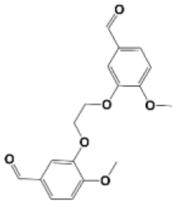	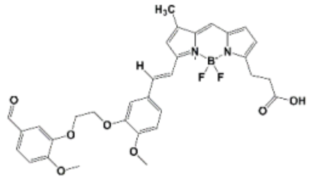	604.22	627.21[b]	8.02	92
BAA-74b	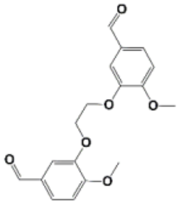	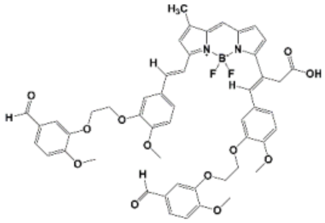	916.32	897.32[a]	7.01	94
BAA-75a	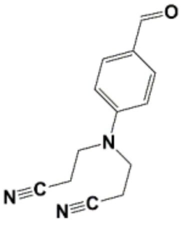	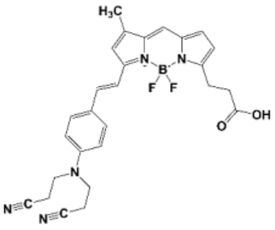	501.21	482.22[a]	10.99	>99
BAA-75b	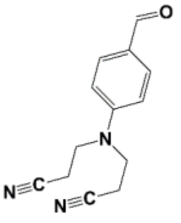	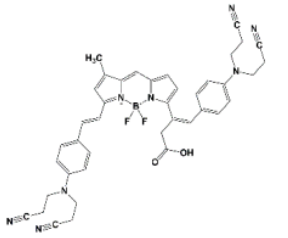	710.31	711.32	6.38	94

Product	Aromatic Aldehyde	Final Structure	m/z calc	m/z exp	RT (min)	Purity (%)
BAA-76a	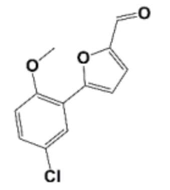	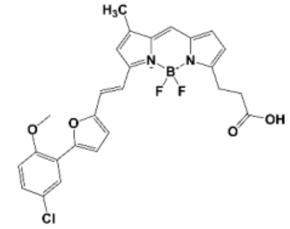	510.13	491.13[a]	90.31	98
BAA-77a	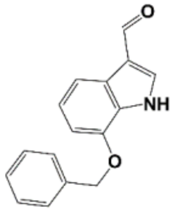	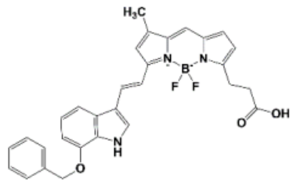	525.2	506.21[a]	8.52	99
BAA-78a	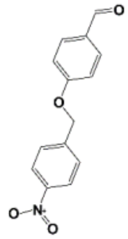	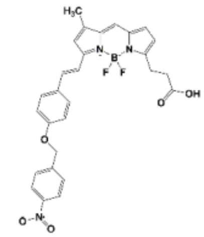	531.18	512.18[a]	8.72	>99
BAA-78b	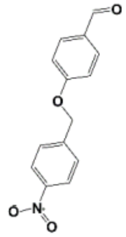	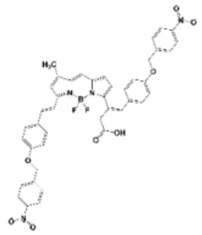	770.24	771.24	7.73	98
BAA-79a	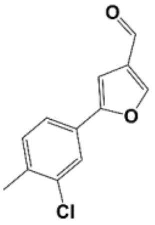	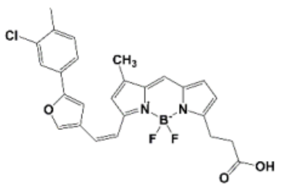	494.14	457.13[d]	9.421	91

LCMS Conditions: A: H_2O-CH_2O_2: 99.9:0.1, B: ACN-CH_2O_2: 99.9:0.1; gradient 5% B to 30% B (1 min), gradient 30% B to 95% B (4.5 min), isocratic 95% B (4.5 min), flowrate: 0.4 ml/min. ESI (+) *m/z* signal found correspond to (M+H) except *a:* (M-F), *b:* (M+Na), *c:* (M+NH_4), *d*: (M-2F+H), *e*: (M-2Cl+H), and *f*: (M-Cl); *g:* Cl replaced by pyrrolidine during synthesis reaction. Purity was determined by integration of all the LCMS absorbance peaks at 254 nm.

<u>Table S2</u>: *Spectroscopic and photochemical properties of BAA library.*

Product	λmax ABS (nm)	λmax EM (nm)	Stokes Shift (nm)	Φ_{fl}	FWHM
BAA-1b	580	597	17	0.62	40
BAA-2a	601	635	34	0.62	57
BAA-3a	569	579	10	0.61	31
BAA-3b	574	587	13	0.70	34
BAA-4a	581	600	19	0.43	56
BAA-5a	592	615	23	0.83	47
BAA-6a	609	643	34	0.18	64
BAA-7a	582	600	18	0.29	63
BAA-7b	581	600	19	0.43	56
BAA-8a	594	623	29	0.50	61
BAA-9a	569	579	10	0.35	32
BAA-10b	632	714	82	0.01	102
BAA-11a	575	589	14	0.86	33
BAA-12a	589	616	27	0.10	88
BAA-13a	576	586	10	0.19	44
BAA-14a	594	607	13	0.44	70
BAA-15a	581	597	16	0.42	39
BAA-16a	552	610	58	0.22	78
BAA-17a	579	591	12	0.59	42
BAA-18a	577	593	16	0.65	37
BAA-19a	582	595	13	0.64	35
BAA-20a	577	585	8	0.15	31
BAA-21a	585	611	26	0.51	46
BAA-21b	588	608	20	0.54	50
BAA-22a	585	602	17	0.39	49
BAA-22b	582	601	19	0.27	46
BAA-23a	592	603	11	0.23	30
BAA-23b	580	588	8	0.19	32
BAA-24b	570	579	9	0.29	32
BAA-25a	571	580	9	0.19	32
BAA-25b	572	580	8	0.18	32
BAA-26b	582	604	22	0.30	51
BAA-27b	575	588	13	0.33	35
BAA-28a	480	592	112	0.04	52
BAA-28b	580	598	18	0.58	43
BAA-29b	569	580	11	0.13	37
BAA-30a	607	643	36	0.43	58

Product	λmax ABS (nm)	λmax EM (nm)	Stokes Shift (nm)	Φ_{fl}	FWHM
BAA-31b	576	588	12	0.28	34
BAA-32a	574	584	10	0.23	32
BAA-33a	575	587	12	0.15	35
BAA-34b	570	581	11	0.96	31
BAA-35b	571	580	9	0.25	32
BAA-36b	579	592	13	0.35	37
BAA-37a	568	578	10	0.54	55
BAA-37b	569	578	9	0.55	30
BAA-38a	579	581	2	0.07	33
BAA-39a	606	657	51	0.19	79
BAA-40b	583	601	18	0.26	45
BAA-41b	582	602	20	0.36	47
BAA-42a	570	580	10	0.36	30
BAA-43a	569	579	10	0.45	31
BAA-44b	582	600	18	0.28	43
BAA-45b	570	579	9	0.50	30
BAA-45a	571	580	9	0.22	31
BAA-46b	572	585	13	0.61	33
BAA-47a	589	611	22	0.33	47
BAA-48a	603	641	38	0.45	60
BAA-49b	571	580	9	0.14	33
BAA-50a	571	581	10	0.28	33
BAA-50b	572	581	9	0.12	34
BAA-51a	594	607	13	0.06	42
BAA-52a	577	585	8	0.04	51
BAA-53a	570	581	11	0.44	30
BAA-54a	573	581	8	0.04	46
BAA-55a	576	592	16	0.75	35
BAA-55b	575	591	16	0.75	38
BAA-56a	585	595	10	0.91	32
BAA-56b	586	597	11	0.15	35
BAA-57a	574	590	16	0.75	45
BAA-57b	575	591	16	0.75	38
BAA-58a	572	582	10	0.70	30
BAA-59a	571	581	10	0.53	30
BAA-60b	588	604	16	0.47	37
BAA-61a	573	587	14	>0.99	35
BAA-62a	579	594	15	0.68	33
BAA-63a	592	607	15	0.43	39

Product	λmax ABS (nm)	λmax EM (nm)	Stokes Shift (nm)	Φ_{fl}	FWHM
BAA-64a	576	593	17	0.80	37
BAA-65a	606	622	16	0.21	54
BAA-66a	587	598	11	0.28	34
BAA-67a	587	598	11	0.15	61
BAA-68a	585	595	10	0.17	30
BAA-69a	577	589	12	0.85	30
BAA-70a	580	588	8	0.17	33
BAA-71a	580	589	9	0.19	32
BAA-72a	580	590	10	0.40	31
BAA-73a	579	593	14	0.55	46
BAA-74a	584	602	18	0.15	59
BAA-74b	583	600	17	0.22	51
BAA-75a	614	663	49	0.08	91
BAA-75b	605	660	55	0.18	91
BAA-76a	613	634	21	0.21	55
BAA-77a	603	634	31	0.50	61
BAA-78a	581	592	11	0.19	46
BAA-78b	579	593	14	0.37	39
BAA-79a	606	630	24	0.63	49

Table S3: *Organelle specificity of BAA Library in fixed U2OS cells.*

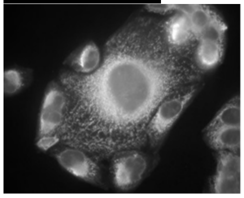
BAA-1b

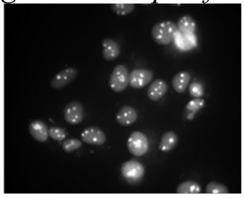
BAA-2a

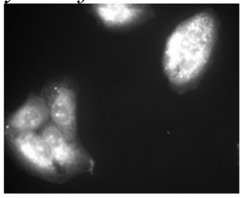
BAA-3a

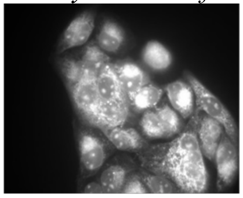
BAA-3b

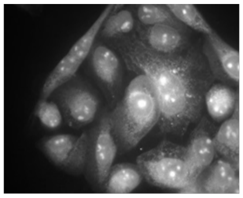
BAA-4a

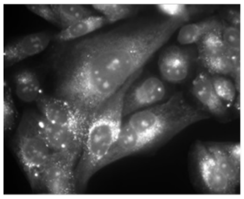
BAA-5a

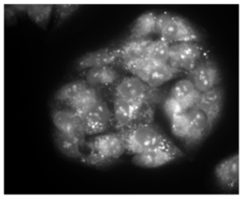
BAA-6a

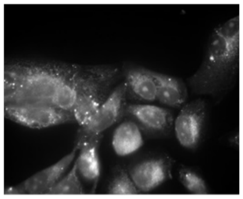
BAA-7a

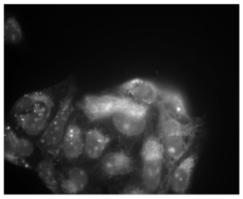
BAA-7b

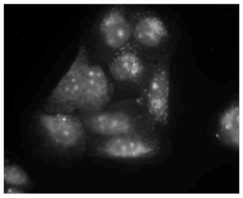
BAA-8a

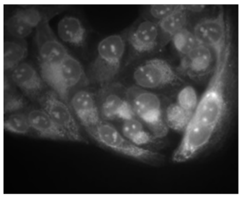
BAA-9a

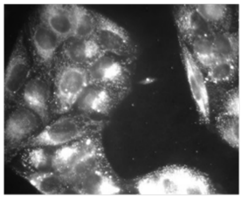
BAA-10b

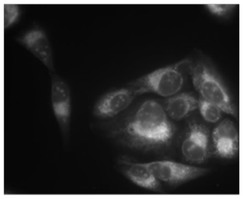
BAA-11a

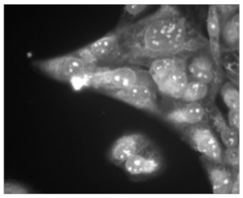
BAA-12a

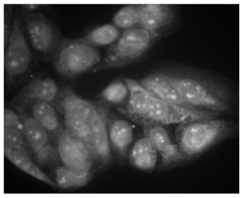
BAA-13a

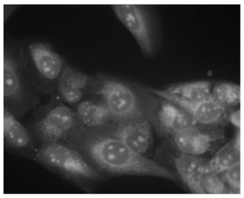
BAA-14a

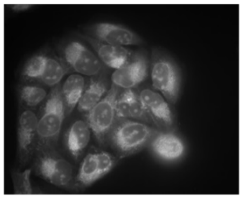
BAA-15a

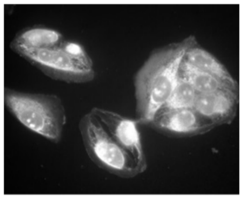
BAA-16a

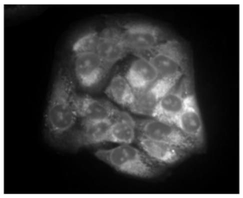
BAA-17a

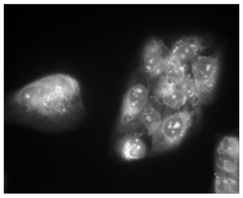
BAA-18a

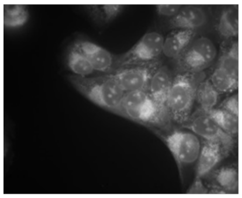
BAA-19a

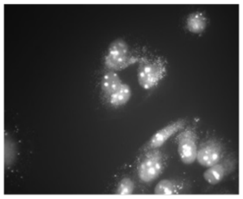
BAA-20a

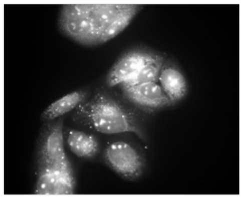
BAA-21a

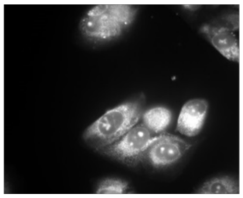
BAA-21b

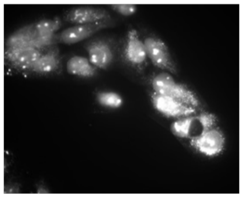
BAA-22a

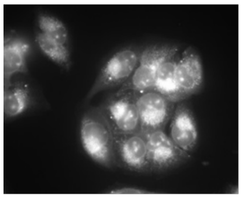
BAA-22b

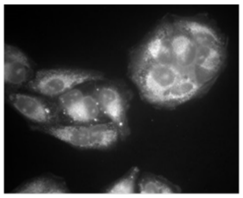
BAA-23a

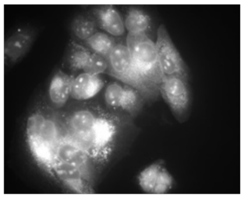
BAA-23b

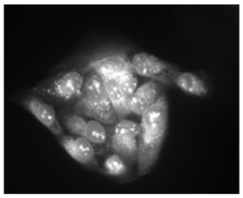
BAA-24b

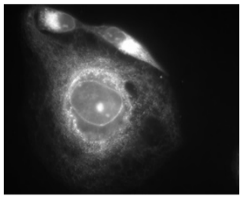
BAA-25a

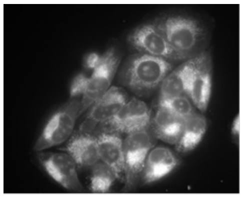
BAA-25b

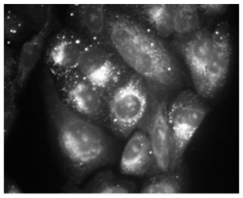
BAA-26b

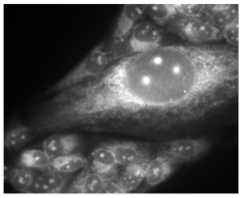
BAA-27b

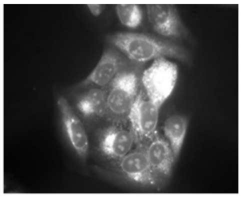
BAA-28a

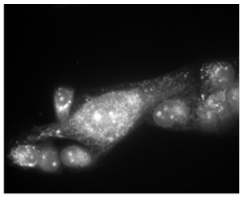
BAA28-b

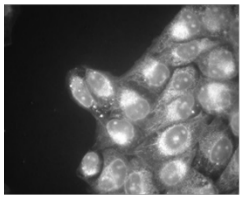
BAA-29b

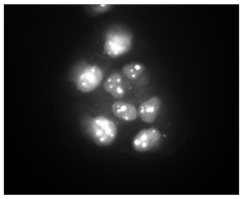
BAA-30a

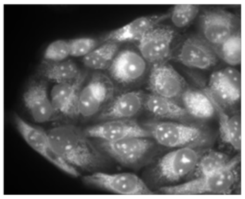
BAA-31b

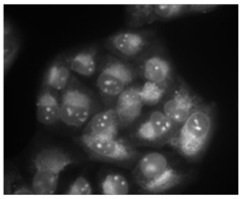
BAA-32a

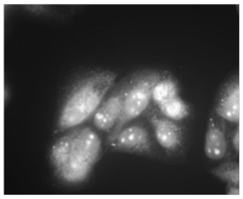
BAA-33a

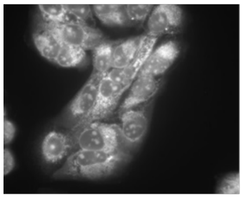
BAA-34b

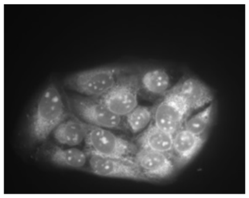
BAA-35b

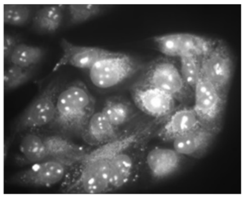
BAA-36b

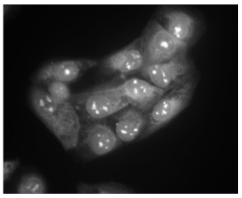
BAA-37a

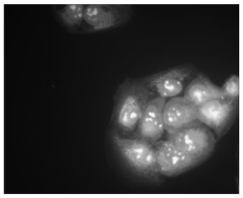
BAA-37b

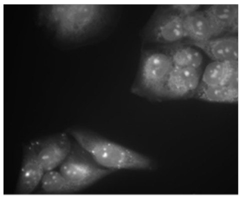
BAA-38a

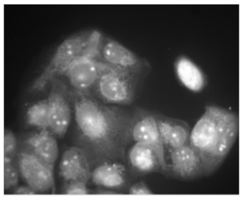
BAA-39a

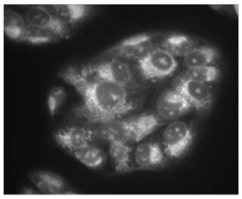
BAA-40b

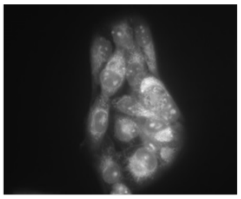
BAA-41b

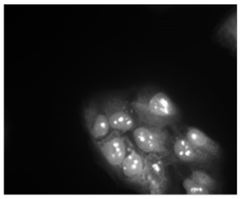
BAA-42a

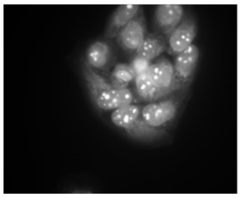
BAA-43a

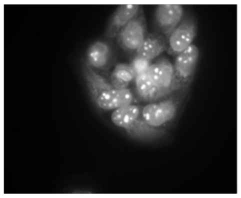
BAA-44b

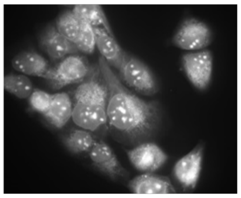
BAA-45a

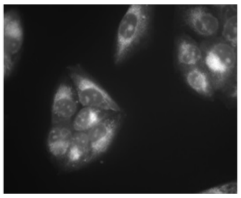
BAA-45b

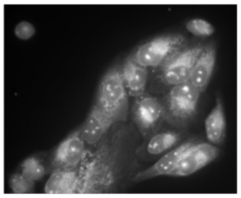
BAA-46b

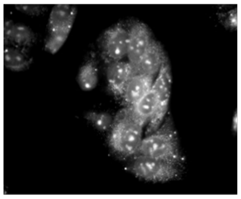
BAA-47a

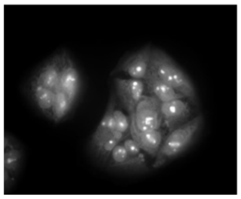
BAA-48a

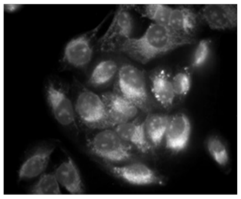
BAA-49b

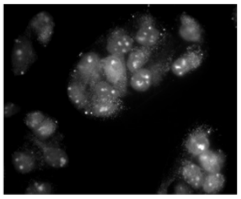
BAA-50a

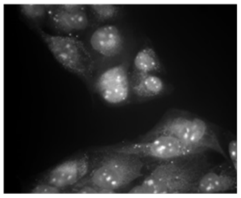
BAA-50b

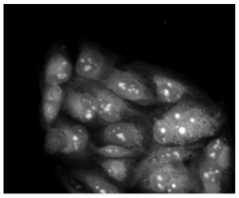
BAA-51a

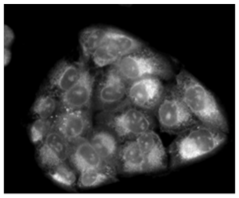
BAA-52a

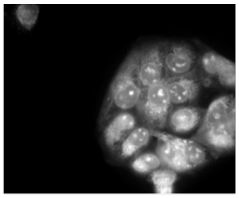
BAA-53a

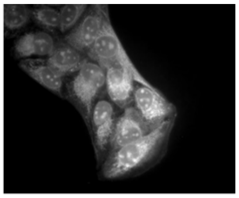
BAA-54a

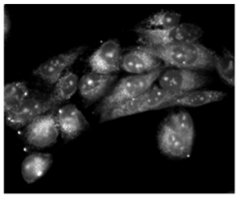
BAA-55a

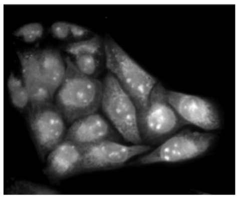
BAA-55b

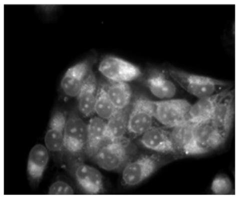
BAA-56a

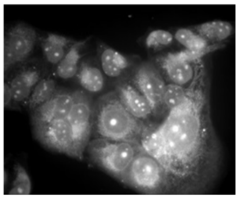
BAA-56b

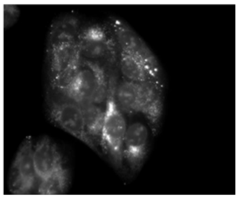
BAA-57a

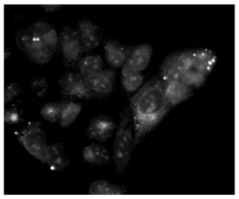
BAA-57b

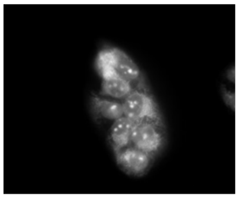
BAA-58a

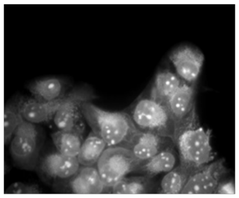
BAA-59a

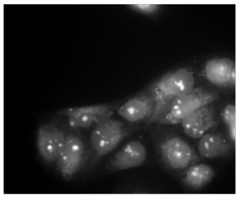
BAA-60b

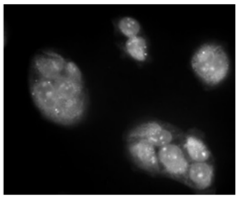
BAA-61a

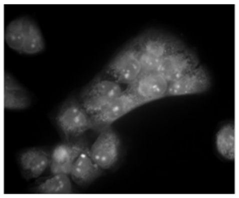
BAA-62a

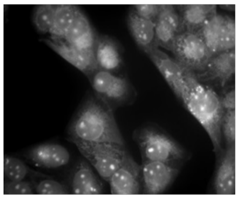
BAA-63a

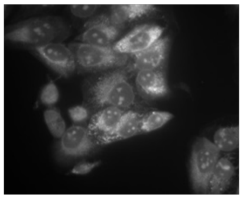
BAA-64a

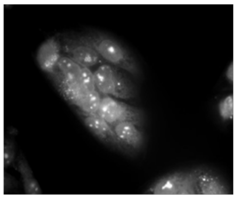
BAA-65a

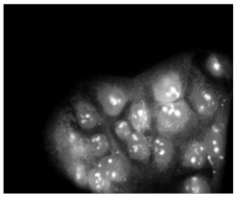
BAA-66a

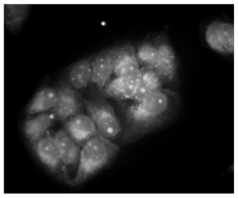
BAA-67a

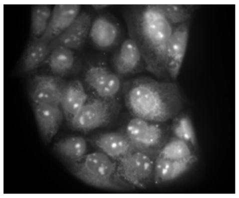
BAA-68a

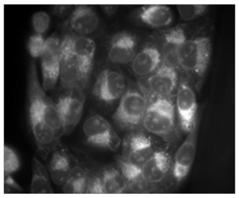
BAA-69a

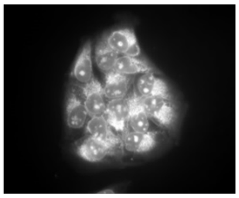
BAA-70a

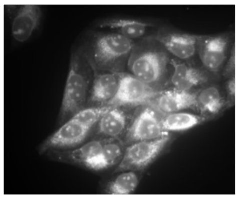
BAA-71a

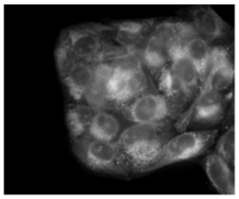
BAA-72a

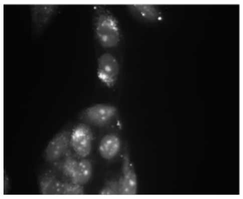
BAA-73a

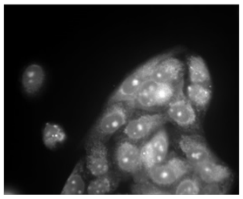
BAA-74a

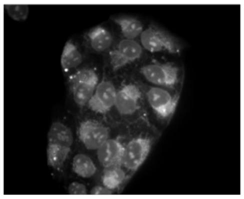
BAA-74b

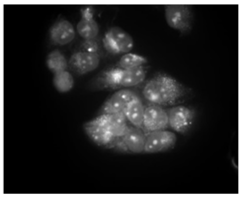
BAA-75a

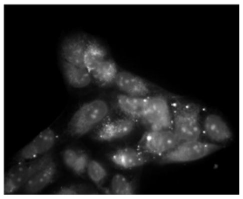
BAA-75b

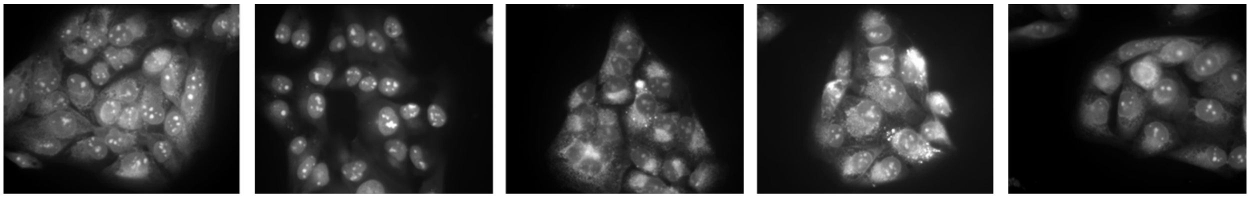

BAA-76a BAA-77a BAA-78a BAA-78b BAA-79a

Images are 168x135 µm and shown with contrast optimized for each dye to show cellular structure localization.

<u>Table S4</u>: *Molecular properties, organelle specificity, and SBR for the BAA library.*

Product	LogD @ pH 7.4	Rotatable Bonds	H Bond Donor	H Bond Acceptor	Polar Surface Area	pK_a	Cell Organelle Specificity	Signal to Background Ratio
BAA-1b	4.20	8	1	6	89.48	3.99	c	4.52
BAA-2a	0.54	5	2	4	61.03	4.54	n	1.71
BAA-3a	3.72	10	1	6	82.16	4.25	c, v	2.19
BAA-3b	0.92	7	1	4	63.70	3.55	v	1.41
BAA-4a	-0.04	6	1	4	51.72	4.16	c,v	1.26
BAA-5a	1.24	12	1	8	100.62	4.25	v	2.74
BAA-6a	0.29	6	2	3	70.26	4.54	c,v	1.06
BAA-7a	0.40	7	1	4	63.70	4.25	v	1.73
BAA-7b	2.68	10	1	6	82.16	4.12	c,v	1.89
BAA-8a	1.19	6	1	3	54.47	4.25	v	1.91
BAA-9a	-0.24	7	3	5	94.93	4.25	c,v	1.63
BAA-10b	4.64	12	1	4	51.72	4.06	v	1.17
BAA-11a	2.90	8	1	3	54.47	4.25	c	4.08
BAA-12a	0.34	8	1	4	71.02	4.25	c	1.89
BAA-13a	0.32	6	1	3	54.47	4.25	c	1.83
BAA-14a	2.00	7	1	2	45.24	4.25	c	1.41
BAA-15a	1.23	8	1	3	54.47	4.25	c,v	4.28
BAA-16a	1.84	5	1	2	45.24	4.25	c	3.01
BAA-17a	2.12	6	1	2	45.24	4.25	c	2.25
BAA-18a	1.96	8	1	3	54.47	4.25	c,v	2.59
BAA-19a	1.54	6	1	2	45.24	4.25	c	0.79
BAA-20a	-0.13	7	1	4	71.02	4.25	v	5.14
BAA-21a	1.71	9	1	4	63.70	4.25	v	6.23
BAA-21b	5.30	14	1	6	82.16	4.04	c	3.52
BAA-22a	1.85	9	1	4	63.70	4.25	c,v	2.07
BAA-22b	5.57	14	1	6	82.16	4.04	c,v	4.27
BAA-23a	2.09	7	1	3	58.13	4.29	v	0.82
BAA-23b	6.06	10	1	4	71.02	4.39	c,v	1.71
BAA-24b	4.73	6	1	2	45.24	3.75	c,v	1.33
BAA-25a	2.52	7	1	2	54.47	4.24	c	1.33
BAA-25b	6.93	10	1	2	63.70	3.95	c	1.52
BAA-26b	4.05	16	1	6	82.16	4.00	v	1.29
BAA-27b	6.65	10	1	2	63.70	4.07	c,v	4.33
BAA-28a	-0.27	6	1	3	63.06	4.26	v	1.86
BAA-28b	1.34	8	1	4	80.88	4.41	v	1.37
BAA-29b	4.83	6	1	2	45.24	3.92	c,v	1.06
BAA-30a	-4.22	8	2	5	85.75	3.52/4.34	n	1.17
BAA-31b	2.44	6	3	4	85.70	4.03	c,v	1.61
BAA-32a	1.82	8	1	4	97.61	4.25	c	3.73
BAA-33a	0.00	6	1	4	79.38	3.99	c,v	1.57
BAA-34b	5.19	8	1	4	71.02	4.60	c	2.57
BAA-35b	5.24	10	1	4	111.28	4.03	c	1.51
BAA-36b	4.85	12	1	6	111.28	4.26	c,v	2.48
BAA-37a	0.81	6	1	3	58.13	4.12	c	2.31
BAA-37b	3.50	8	1	4	71.02	4.07	c	1.42
BAA-38a	0.68	6	1	4	61.37	4.26	c,v	1.50
BAA-39a	0.39	6	1	4	61.37	4.07	c	1.18
BAA-40b	7.06	16	1	6	82.16	4.00	c	1.52

Product	LogD @ pH 7.4	Rotatable Bonds	H Bond Donor	H Bond Acceptor	Polar Surface Area	pK_a	Cell Organelle Specificity	Signal to Background Ratio
BAA-41b	6.23	14	1	6	82.16	4.04	c	1.94
BAA-42a	3.30	8	1	3	71.54	4.25	c	1.69
BAA-43a	2.78	8	1	3	71.54	4.25	c	2.16
BAA-44b	7.06	16	1	6	82.16	4.04	c	3.26
BAA-45b	7.12	12	1	4	63.70	4.25	c	2.34
BAA-45a	2.62	8	1	3	54.47	4.19	c	5.01
BAA-46b	5.30	14	1	6	82.16	4.12	c	6.52
BAA-47a	0.62	9	1	4	63.70	4.25	v	5.90
BAA-48a	2.06	8	2	3	70.26	4.53	c	1.77
BAA-49b	0.50	12	1	8	129.74	3.86	c	1.00
BAA-50a	0.52	6	1	3	63.06	4.25	v	1.97
BAA-50b	2.92	8	1	4	80.88	4.29	c,v	1.50
BAA-51a	-0.02	7	2	4	83.57	4.25	c	10.02
BAA-52a	0.72	8	1	4	63.70	4.25	c	0.77
BAA-53a	-0.07	7	1	4	63.70	4.25	c,v	0.63
BAA-54a	1.10	5	1	2	45.24	4.25	c,v	0.69
BAA-55a	0.41	6	1	3	63.06	4.25	v	2.61
BAA-55b	2.69	8	1	4	80.88	4.20	c	1.54
BAA-56a	0.62	6	1	5	58.13	4.25	c,v	1.63
BAA-56b	3.13	8	1	6	71.02	4.77	c,v	1.44
BAA-57a	0.87	6	1	3	63.06	4.25	v	1.67
BAA-57b	3.63	8	1	4	80.88	4.17	v	3.63
BAA-58a	2.27	8	1	3	71.54	4.25	c	19.25
BAA-59a	0.26	7	1	5	92.27	4.25	c	1.77
BAA-60b	4.60	8	1	2	45.24	4.77	v	6.04
BAA-61a	1.02	9	3	5	94.93	4.25	c,v	4.57
BAA-62a	0.13	5	1	3	58.13	4.16	c,v	1.79
BAA-63a	1.88	5	1	3	58.13	4.24	c	5.92
BAA-64a	1.37	6	2	3	73.92	4.25	c,v	1.95
BAA-65a	0.97	6	1	2	58.38	4.25	c,v	1.04
BAA-66a	1.07	6	1	3	58.13	4.25	c,v	0.94
BAA-67a	2.10	8	1	3	58.13	4.26	v	1.94
BAA-68a	0.98	6	1	3	58.13	4.26	v	1.86
BAA-69a	2.24	6	1	3	58.13	4.26	c,v	0.96
BAA-70a	2.09	7	1	3	58.13	4.26	c,v	2.08
BAA-71a	2.89	8	1	4	67.36	4.26	c,v	1.88
BAA-72a	1.49	6	1	3	58.13	4.26	c,v	5.15
BAA-73a	0.95	8	1	3	54.47	4.25	v	1.90
BAA-74a	0.97	13	1	7	99.23	4.25	c,v	2.70
BAA-74b	3.82	22	1	12	153.22	4.00	c,v	2.25
BAA-75a	-0.18	10	1	5	96.06	4.25	v	3.83
BAA-75b	1.52	16	1	8	146.88	4.27	v	1.34
BAA-76a	1.10	7	1	3	67.61	4.25	c,v	1.58
BAA-77a	2.06	8	2	3	70.26	4.28	n	3.21
BAA-78a	1.92	9	1	5	97.61	4.25	c,v	6.81
BAA-78b	5.71	14	1	8	149.98	4.12	c,v	2.44
BAA-79a	2.06	6	1	2	58.38	4.25	c	3.43

BAA compounds showed localization to cytosol (c), vesicles (v), and nucleolar (n) regions.

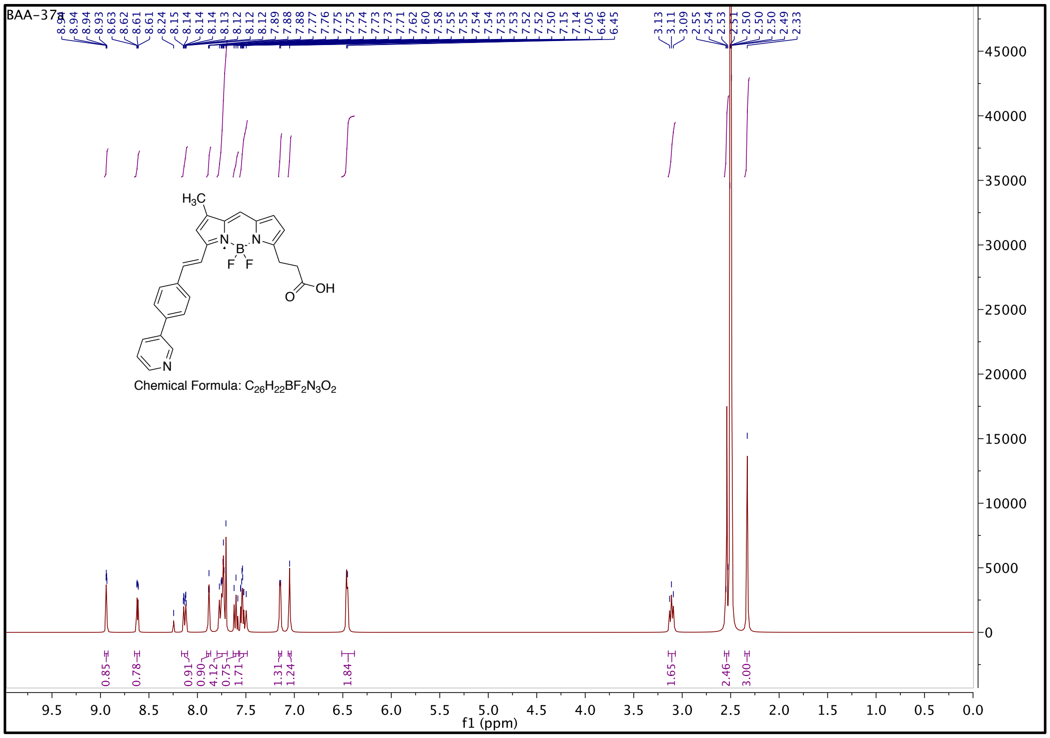

Figure S1: ^{1}H NMR spectrum of compound **BAA-37a**: (400 MHz, DMSO-d_6) δ 8.94 (dd, J = 2.5, 0.9 Hz, 1H), 8.62 (dd, J = 4.8, 1.6 Hz, 1H), 8.13 (ddd, J = 8.0, 2.5, 1.6 Hz, 1H), 7.88 (t, J = 1.7 Hz, 1H), 7.80 – 7.69 (m, 4H), 7.60 (t, J = 7.7 Hz, 1H), 7.56 – 7.49 (m, 2H), 7.15 (d, J = 4.0 Hz, 1H), 7.05 (s, 1H), 6.46 (d, J = 4.0 Hz, 2H), 3.11 (t, J = 7.8 Hz, 2H), 2.53 (t, J = 7.8 Hz, 2H), 2.33 (s, 3H).

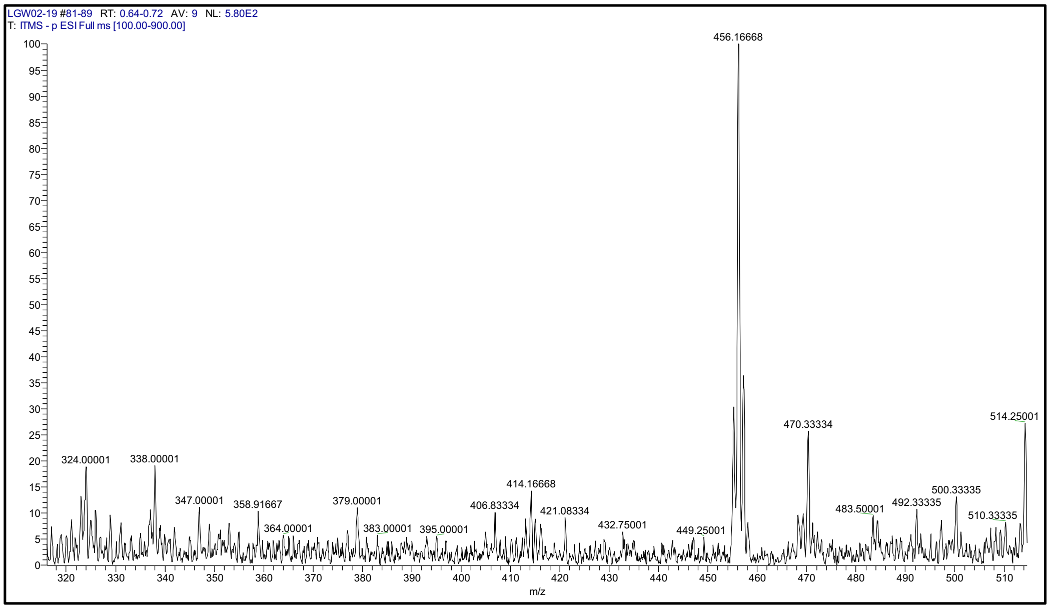

Figure S2: HR ESI negative mode spectrum of compound **BAA-37a**. HRMS-ESI (m/z): $[M-H]^-$ calculated for $C_{26}H_{21}BF_2N_3O_2^-$: 456.17004; found: 456.16668 (7.4 ppm).

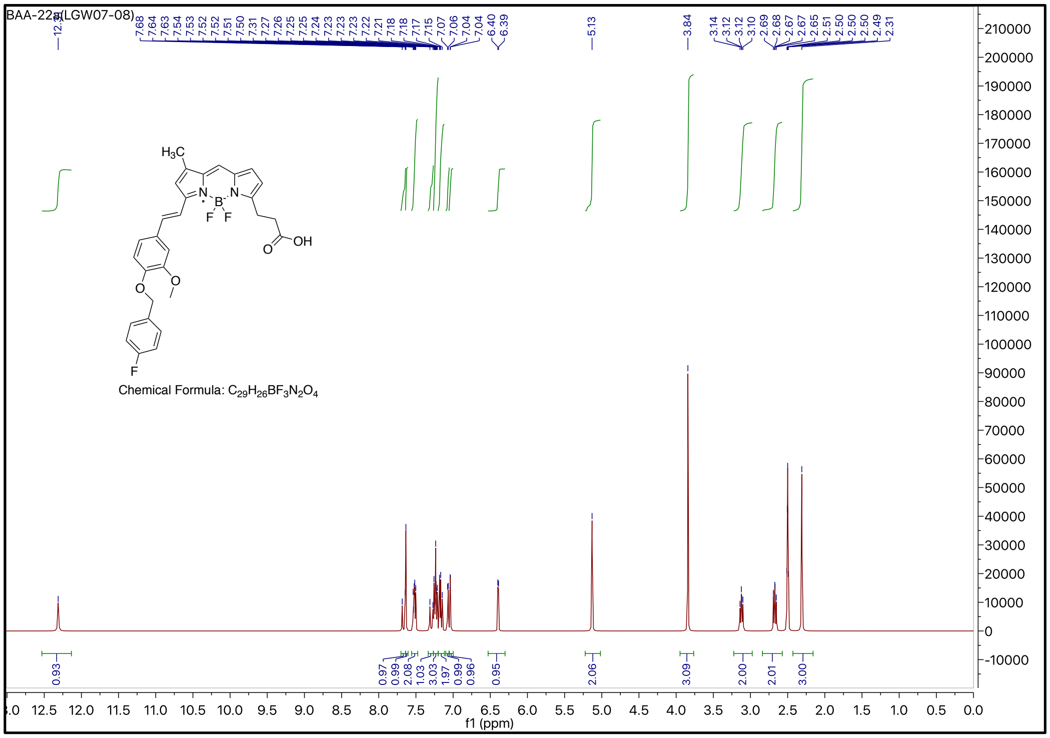

<u>Figure S3</u>: ^{1}H NMR spectrum of compound **BAA-22a**: (400 MHz, DMSO-d_6) δ 12.31 (s, 1H), 7.66 (d, J = 16.3 Hz, 1H), 7.63 (s, 1H), 7.56 – 7.48 (m, 2H), 7.29 (d, J = 16.3 Hz, 1H), 7.26 – 7.20 (m, 3H), 7.20 – 7.12 (m, 2H), 7.07 (d, J = 4.0 Hz, 1H), 7.04 (s, 1H), 6.39 (d, J = 3.9 Hz, 1H), 5.13 (s, 2H), 3.84 (s, 3H), 3.12 (t, 2H), 2.67 (t, 2H), 2.31 (s, 3H).

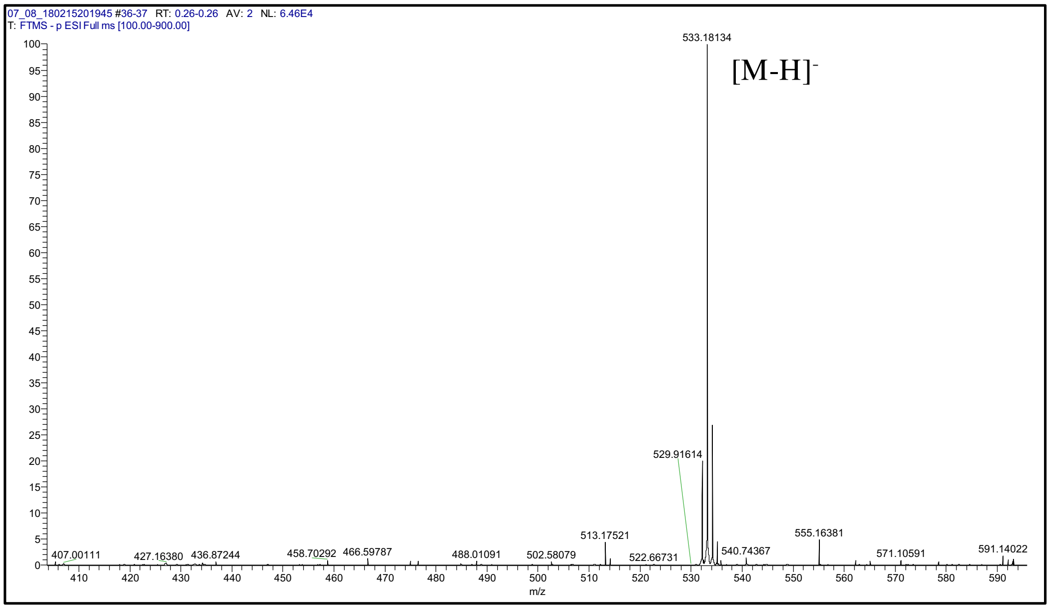

<u>Figure S4</u>: HR ESI negative mode spectrum of compound **BAA-22a**. HRMS-ESI (m/z): $[M-H]^-$ calculated for $C_{29}H_{25}BF_3N_2O_4^-$: 533.18650; found: 533.18134 (9.7 ppm).

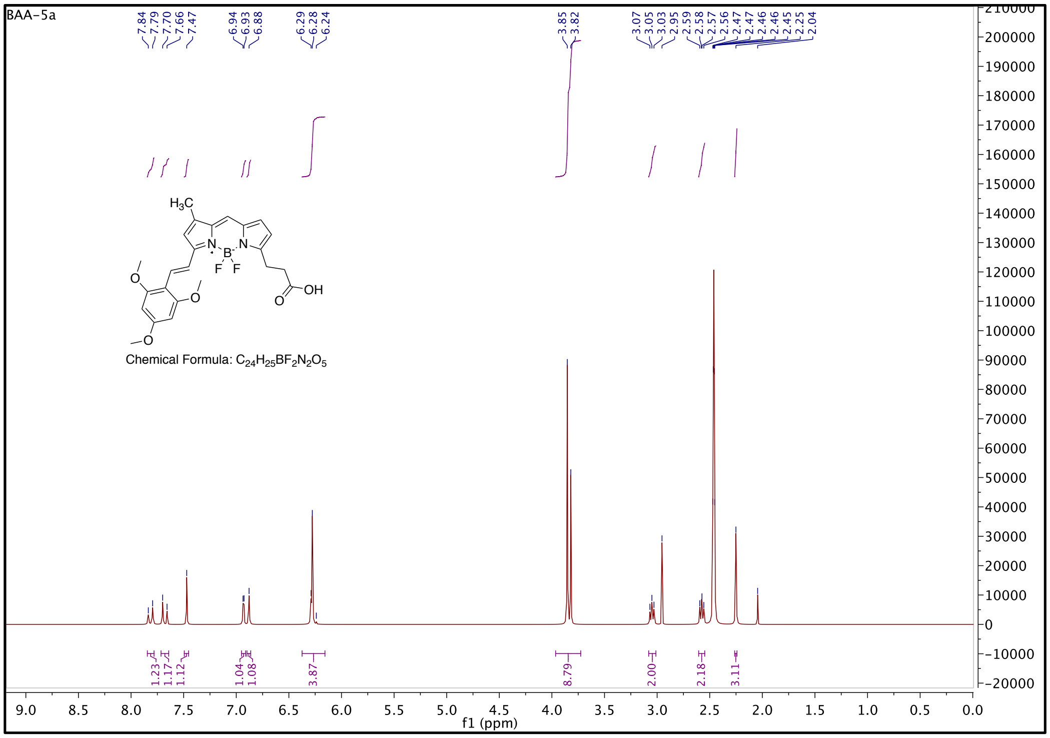

<u>Figure S5</u>: ^{1}H NMR spectrum of compound **BAA-5a**: (400 MHz, DMSO-d_6) δ 7.82 (d, J = 16.4 Hz, 1H), 7.68 (d, J = 16.4 Hz, 1H), 7.47 (s, 1H), 6.93 (d, J = 4.0 Hz, 1H), 6.88 (s, 1H), 6.37 – 6.16 (m, 4H), 3.96 – 3.73 (m, 9H), 3.05 (t, J = 7.7 Hz, 2H), 2.58 (t, J = 7.7 Hz, 2H), 2.25 (s, 3H).

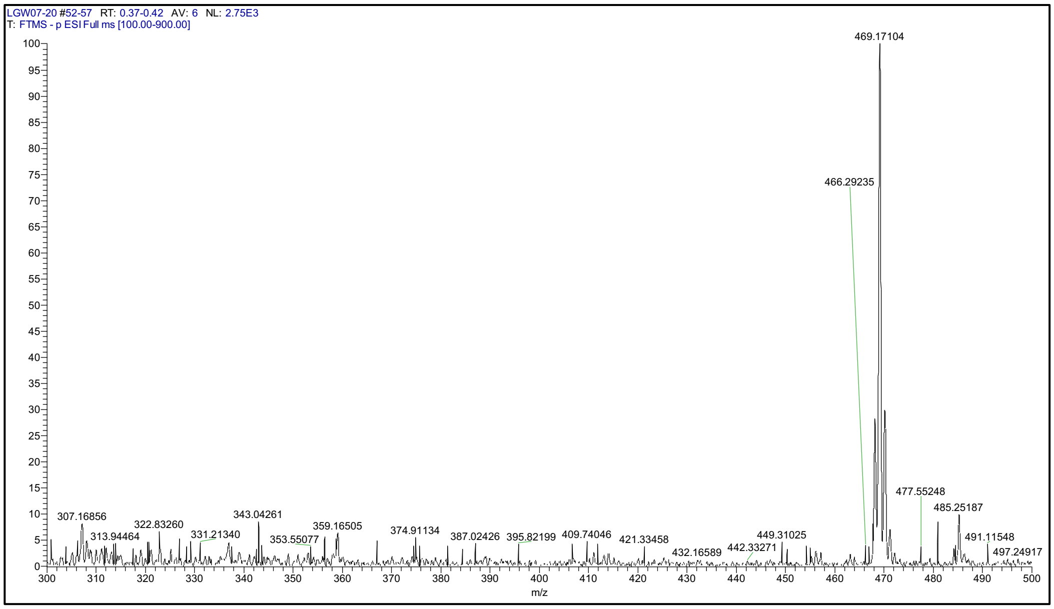

<u>Figure S6</u>: HR ESI negative mode spectrum of compound **BAA-5a**. HRMS-ESI (m/z): $[M\text{-}H]^-$ calculated for $C_{24}H_{24}BF_2N_2O_5^-$: 469.17518; found: 469.17104 (9.7 ppm).

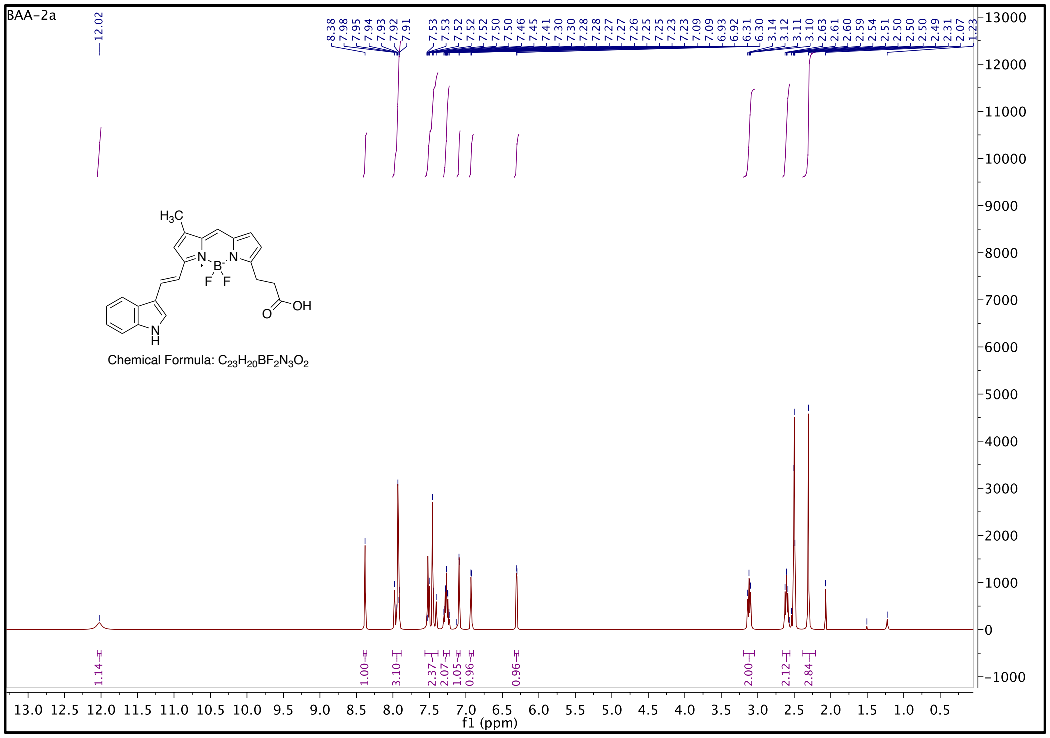

<u>Figure S7</u>: ^{1}H NMR spectrum of compound **BAA-2a**: (400 MHz, DMSO-d_6) δ 12.02 (s, 1H), 8.38 (s, 1H), 8.00 – 7.89 (m, 3H), 7.56 – 7.38 (m, 2H), 7.31 – 7.23 (m, 2H), 7.09 (s, 1H), 6.93 (d, J = 3.9 Hz, 1H), 6.30 (d, J = 3.9 Hz, 1H), 3.12 (t, J = 8.8 Hz, 2H), 2.61 (t, J = 8.8 Hz, 2H), 2.31 (s, 3H).

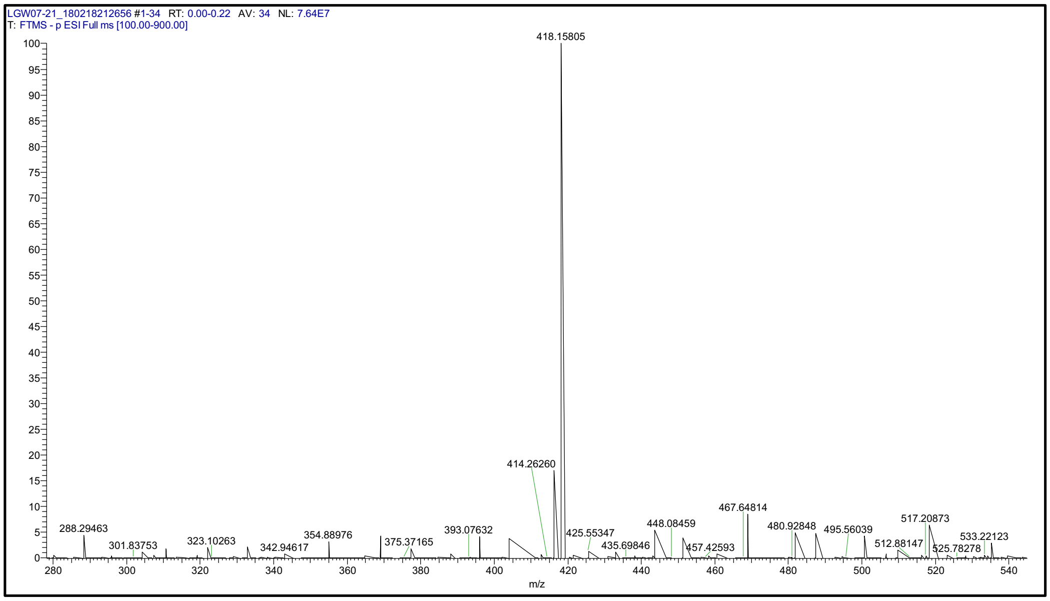

<u>Figure S8</u>: HR ESI negative mode spectrum of compound **BAA-2a**. HRMS-ESI (m/z): $[M-H]^-$ calculated for $C_{23}H_{19}BF_2N_3O_2^-$: 418.15439; found: 418.15805 (8.8 ppm).

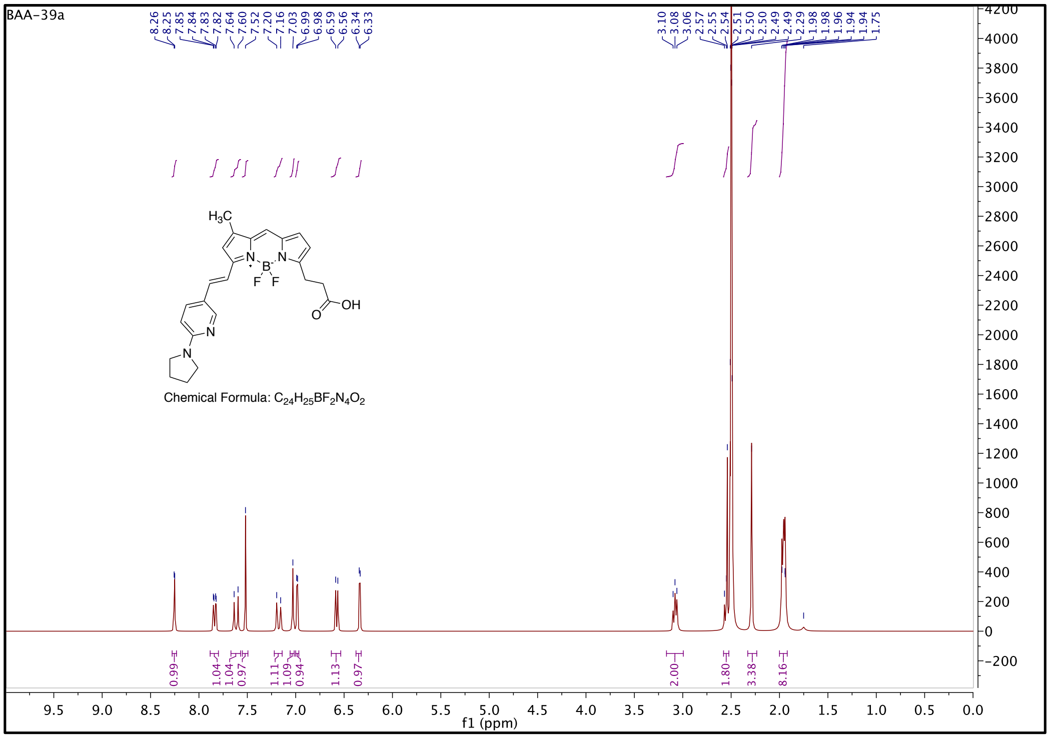

Figure S9: ^{1}H NMR spectrum of compound **BAA-39a**: (400 MHz, DMSO-d_6) δ 8.25 (d, J = 2.5 Hz, 1H), 7.84 (dd, J = 9.0, 2.4 Hz, 1H), 7.62 (d, J = 16.2 Hz, 1H), 7.52 (s, 1H), 7.18 (d, J = 16.2 Hz, 1H), 7.03 (s, 1H), 6.99 (d, J = 3.9 Hz, 1H), 6.58 (d, J = 9.0 Hz, 1H), 6.34 (d, J = 4.0 Hz, 1H), 3.08 (t, J = 11.8 Hz, 2H), 2.55 (t, J = 11.8 Hz, 2H), 2.26 (s, 3H), 2.00 – 1.92 (m, 8H).

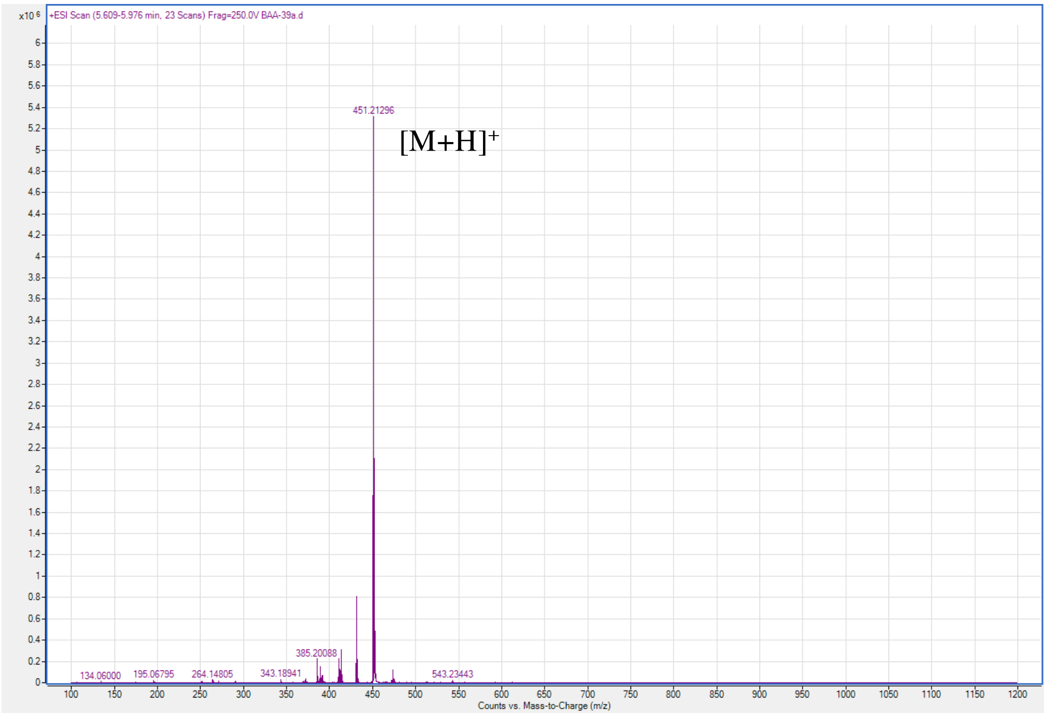

Figure S10: HR ESI negative mode spectrum of compound **BAA-39a**. HRMS-ESI (m/z): $[M+H]^+$ calculated for $C_{24}H_{26}BF_2N_4O_2^+$: 451.21114; found: 451.21296 (4.0 ppm).

A. BAA-37a

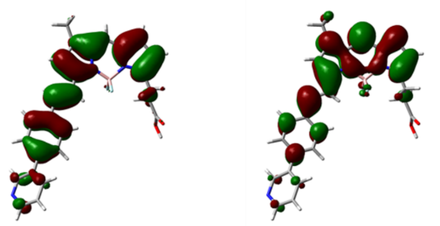

B. BAA-22a

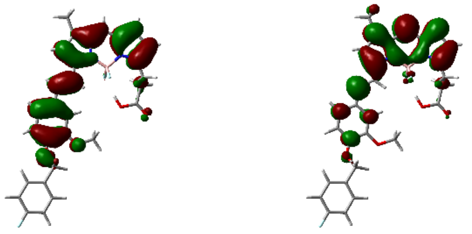

C. BAA-5a

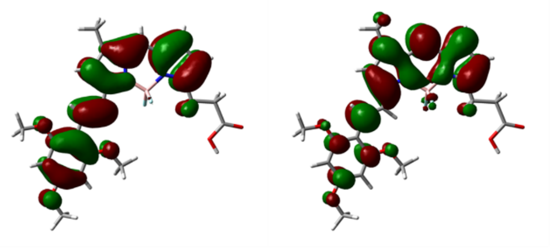

D. BAA-2a

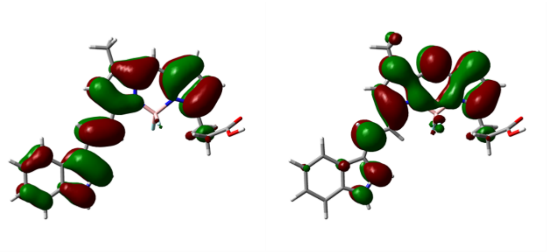

E. BAA-39a

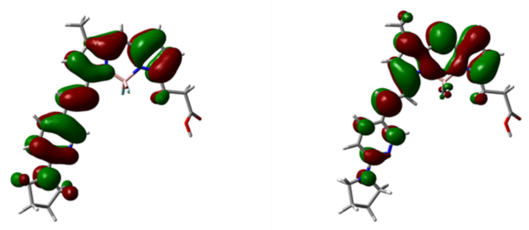

<u>Figure S11</u>: The HOMO and LUMO molecular orbitals are shown on the left and right, respectively of the five selected BAA compounds for immunofluorescence imaging. **A. BAA-37a**, **B. BAA-22a**, **C. BAA-5a**, **D. BAA-2a** and **E. BAA-39a**.
